# Coupling two enzymes into a tandem nanoreactor utilizing a hierarchically structured MOF†
†Electronic supplementary information (ESI) available. See DOI: 10.1039/c6sc01438k



**DOI:** 10.1039/c6sc01438k

**Published:** 2016-07-14

**Authors:** Xizhen Lian, Ying-Pin Chen, Tian-Fu Liu, Hong-Cai Zhou

**Affiliations:** a Department of Chemistry , Texas A&M University , College Station , Texas 77842-3012 , USA . Email: zhou@chem.tamu.edu; b Department of Materials Science and Engineering , Texas A&M University , College Station , Texas 77843 , USA

## Abstract

A hierarchical porous metal–organic framework (MOF), PCN-888, containing three types of cavities was utilized to couple two enzymes into a tandem nanoreactor. The largest cavity (6.2 nm) can only accommodate one molecule of glucose oxidase (GOx). The intermediate cavity (5.0 nm) can accommodate one and only one molecule of horseradish peroxidase (HRP). The small cavity (2.0 nm) has sufficient size for neither GOx nor HRP, and remains open as a substrate diffusion pathway. The coupling of the two enzymes can only be achieved through a unique stepwise encapsulation with a specific order (GOx first, followed by HRP). The tandem nanoreactor shows excellent catalytic performances and negligible enzyme leaching. Its catalytic activity is well maintained within several catalytic cycles. Moreover, PCN-888 can provide protection to the encapsulated enzymes against trypsin digestion, indicating the *in vitro* and *in vivo* stability of the nanoreactor.

## Abstract 2

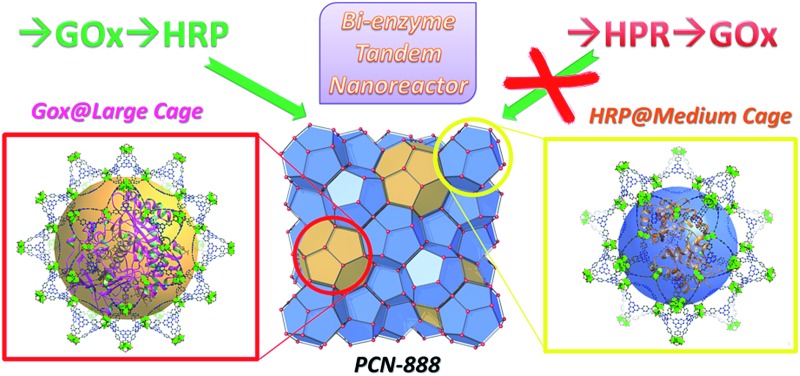
A hierarchically structured MOF is utilized to couple two enzymes in a tandem manner. A stepwise encapsulation with a specific order is the only way to achieve this goal.

## Introduction

During the course of evolution, nature has developed an ingenious series of multi-enzymatic systems to catalyze cascade reactions in the microenvironment of a cell.^1^ In mitochondria, for instance, eight enzymes are involved in the citric acid cycle, catalyzing a controlled metabolism of sugars, fats and proteins and a highly effective production of ATP with minimal consumption of energy.^1,2^ Inspired by the biosynthetic efficiencies of nature and in the search for more sustainable alternatives to today's ways of producing chemicals, scientists have tried to couple multiple enzymes for synthesis in a cascade manner.^1,3^ Specifically, in order to prevent the fragile enzymatic catalytic processes from undesired or toxic conditions, various enzymatic cascade nanoreactors have been developed based on porous materials such as phospholipid liposomes, polymersomes and mesoporous silica.^4–8^ Although they demonstrated enzymatic reactivity, their catalytic performances were still far from satisfactory for any real applications. A major problem is that a high enzyme encapsulation capacity, which is critical to fulfill their desired functions, is not achievable in these materials.^5^ Furthermore, the weak mechanical stability of liposomes, the poor permeability of the polymersome membrane and the severe enzyme leaching from mesoporous silica materials in cyclic uses are limiting factors due to the nature of the materials.^4,9^ Therefore, searching for more promising materials for enzyme encapsulation and coupling is highly desirable.

**Scheme 1:**
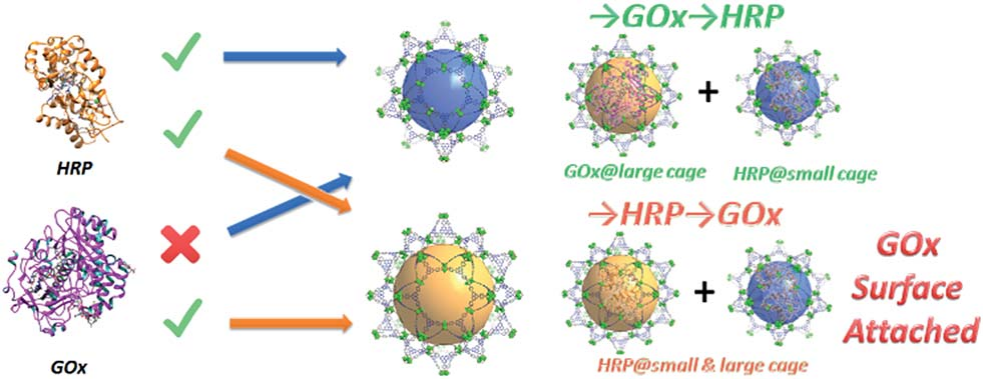
Graphic representation of the results of the stepwise encapsulation of GOx and HRP with different orders.

Metal–organic frameworks (MOFs) are an emerging class of porous materials with a vast application potential.^10–15^ It has been established that cage-containing MOFs (cageMOFs) always act as efficient molecular traps affording strong interactions between the framework and the encapsulated moieties;^16,18–24^ especially in one case single enzyme encapsulation was achieved in a hierarchical mesoporous MOF, leading to a record high enzyme encapsulation capacity and excellent catalytic performances.^9^ In this work the same design approach is applied to deal with a much more sophisticated problem—coupling two enzymes in a tandem manner with a precise control of the distribution of each enzyme in the nanoreactor. To achieve this aim, a novel hierarchical mesoporous MOF, PCN-888, which contains three types of cages with different sizes, is rationally designed and synthesized. The largest cage of PCN-888 accommodates one GOx while the medium cage accommodates one HRP. A stepwise encapsulation with a specific encapsulation order (GOx first, then HRP) is a key operation to achieve the bi-enzyme coupling in PCN-888 (Scheme 1). The smallest cage is too compact for either enzyme, thus it is left empty as a diffusion pathway for substrates and products. PCN-888 demonstrates not only a very high enzyme loading but also a negligible enzyme leaching, whereas the catalytic activity of the encapsulated enzymes is well maintained. Moreover, this nanoreactor shows a convincing reusability and outstanding stability against the digestion of trypsin, indicating its potential applications for *in vitro* or *in vivo* studies.

**Fig. 1:**
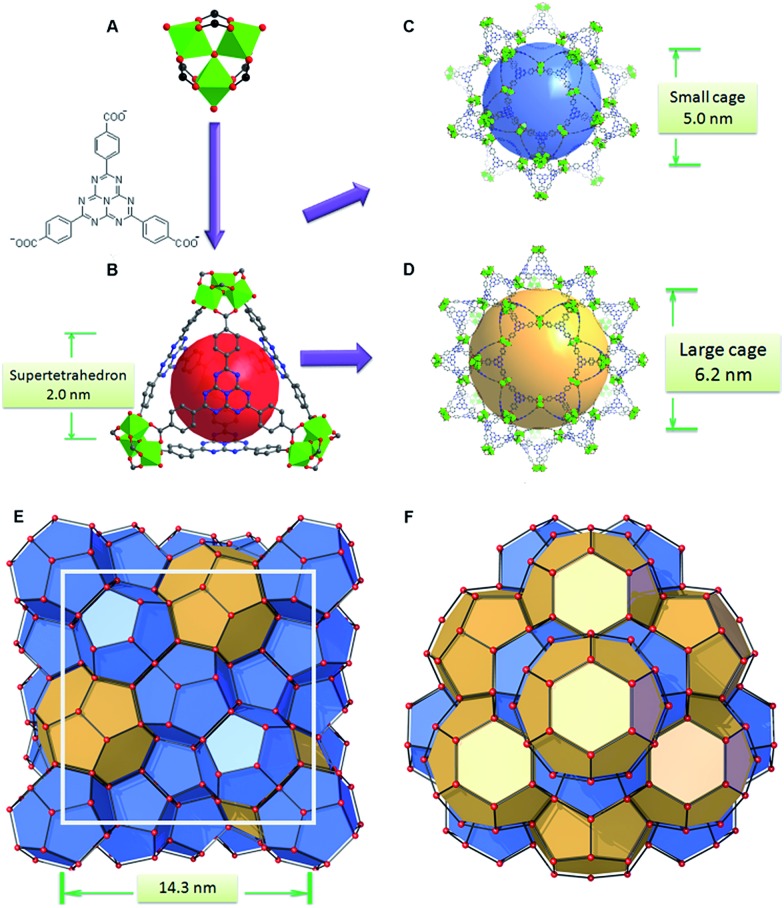
(A) The trimeric cluster and the HTB ligand to construct (B) a super tetrahedron; (C) the small cage and (D) large cage of PCN-888 composed of the super tetrahedron in a vertex sharing manner; (E) **mtn** topology with a cell diameter of 14.3 nm; (F) large cages in a honeycomb-like arrangement viewed from the [111] direction.

**Fig. 2:**
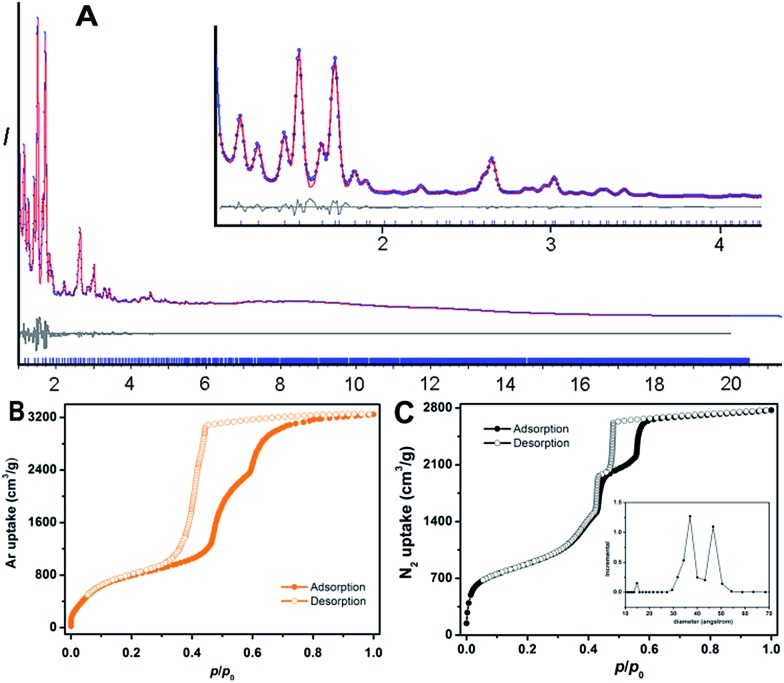
(A) Rietveld refinement patterns of PCN-888 using synchrotron PXRD data (*λ* = 0.72768 Å): observed (blue), calculated (red), and difference (grey) profiles are shown; the tick marks below the curves indicate Bragg positions. The X-ray diffraction pattern between 1.1° and 4.2° is magnified in the inset. The full pattern shows a precise match between the experimental PXRD data and those simulated from the proposed structure. (B) Ar isotherms of PCN-888 measured at 87 K. (C) N_2_ isotherms of PCN-888 measured at 77 K. Pore size distribution obtained from the N_2_ isotherm is displayed in the inset.

PCN-888 was obtained by a solvothermal reaction with AlCl_3_ and a heptazine based tritopic ligand (HTB) at 135 °C. PCN-888 is isoreticular to PCN-333, which was shown earlier by our group.^9^ High-resolution synchrotron powder X-ray diffraction (PXRD) collected at 17-BM, Argonne National Laboratory shows that PCN-888 is cubic with an exceptionally large unit cell length *a* ≈ 143 Å (Fig. 1). PCN-333 crystallizes in the space group *Fd*3*m*, therefore, the same space group was chosen to describe the isoreticular PCN-888. The corresponding structural model of PCN-888, with a formula of [C_54_H_24_N_14_O_16_Al_3_], was simulated based on the reported PCN-333 structure using Material Studio 6.0.^27^ Rietveld refinement was performed to examine the validity of the structure model, which converged to an acceptable *R*_wp_ value of 0.0349, an *R*_exp_ of 0.0583 and an *R*_p_ of 0.0181 (Fig. 2A).

The basic secondary building block of PCN-888 is a super tetrahedron, which consists of an aluminum trimeric cluster Al_3_(μ_3_-O)(OH)(H_2_O)_2_ at the four vertices and HTB ligand located at the four faces, in a vertex sharing manner (Fig. 1B). The HTB ligands experience geodesic bending to form a domed structure in the framework, giving rise to a hierarchical porous structure with three types of mesoporous cages. Besides the super tetrahedral cage mentioned above, there are two additional hierarchal mesoporous cages in the PCN-888 structure. A smaller dodecahedral cage is composed of 20 super tetrahedra connected by vertex sharing with a pentagonal window of 2.5 nm (Fig. 1C). A larger hexacaidecahedral (hexagonal-truncated trapezohedral) cage is surrounded by 28 super tetrahedra with not only the 2.5 nm pentagonal windows, but also hexagonal windows of 3.6 nm (Fig. 1D). The large cages lie in a honeycomb-like arrangement in the [111] projection, as shown in Fig. 1F. The diameter of the inscribed sphere is 2.0 nm for the super tetrahedron cage, 5.0 nm for the dodecahedral cage and 6.2 nm for the hexacaidecahedral cage. The porosity of PCN-888 was determined by Ar adsorption at 87 K and N_2_ adsorption at 77 K. It has a total Ar uptake of 3250 cm^3^ g^–1^ and N_2_ uptake of 2770 cm^3^ g^–1^ (Fig. 2B and C). The Brunauer–Emmett–Teller surface area is over 3700 m^2^ g^–1^. The two steep increases at *p*/*p*_0_ = 0.44 and 0.56 on the adsorption branch of the Ar isotherms correspond to the filling of the dodecahedral and the hexacaidecahedral cages of PCN-888, respectively. The experimental pore volume is 4.0 cm^3^ g^–1^. Such a high porosity is among the most porous materials reported in the literature^25^ and the hierarchical structure indicates that it is appropriate for constructing an enzymatic nanoreactor with an ultrahigh enzyme loading and low leaching.^9,20,21,25^

Only one molecule of GOx (6.0 × 5.2 × 7.7 nm) can fit in the largest cage (6.2 nm) of PCN-888, while HRP (4.0 × 4.4 × 6.8 nm) can be accommodated in both the medium (5.0 nm) and large cages. Therefore, a stepwise encapsulation with the order → GOx → HRP is necessary to precisely control the distribution of GOx and HRP exclusively in the largest and medium cages, respectively. With the above order, PCN-888 demonstrated a GOx uptake capacity of 1.0 g g^–1^ and HRP uptake capacity of 2.0 g g^–1^. The total enzyme encapsulation capacity was 300 wt%, which is the highest among all MOFs.^9,16,17^ In the following discussion the nanoreactor generated with the →GOx → HRP order is named PCN-888-en.

The porosity of PCN-888-en has dramatically decreased due to the residence of enzymes in pores. The total N_2_ uptake of PCN-888-en was reduced to 400 cm^3^ g^–1^ whereas the BET surface area dropped to 147 m^2^ g^–1^. The pore size distribution analysis revealed that the pore size of PCN-888-en is predominantly around 1.4 nm, which corresponds to the cavity of the smallest pore (super tetrahedron), whereas the contributions from the two mesoporous cages of 5.0 nm and 6.2 nm in pristine PCN-888 disappeared. Consequently, we can conclude that the enzymes are residing in the mesoporous cages of PCN-888, while the smallest pore is left empty, which may provide a pathway for the diffusion of substrates into the framework. N_2_ isotherms of GOx@PCN-888 and HRP@PCN-888 were also collected to prove the size selective incorporation of enzymes by different cages. The contribution from the medium cage was present on the pore size analysis pattern of GOx@PCN-888 while the mesoporous porosity was fully occupied in the case of HRP@PCN-888 (ESI, Fig. S10 and S12†).

When the order of enzyme encapsulation was reversed to →HRP → GOx, the HRP uptake was higher (2.5 g g^–1^) whereas the GOx uptake was greatly compromised (0.07 g g^–1^). The reversed order leads to only a surface attachment of GOx, as can be revealed from the negligible uptake amount, indicating that bi-enzyme coupling is not achieved. The nanoreactor generated with the order →HRP → GOx is named PCN-888-enR.

GOx catalyzes the reaction between glucose and molecular oxygen, generating gluconolactone and hydrogen peroxide. Hydrogen peroxide is consumed in the conversion of ABTS to ABTS˙^+^, catalyzed by HRP. The absolute reaction kinetics for this tandem reaction was obtained by monitoring the formation of ABTS˙^+^ at 403 nm by UV-vis spectroscopy, instead of H_2_O_2_, since the rate of reaction catalyzed by GOx is slower than that of HRP.^7^ The catalytic performance of PCN-888-en was first examined. The apparent reaction rate (*k*_cat_), substrate affinity (*K*_m_) and maximum conversion rate (*v*_max_) of PCN-888-en have proved the well maintained catalytic activities of the encapsulated enzymes in PCN-888. The design of only one enzyme residing in each pre-designed cage effectively prevents enzyme aggregation. Moreover, since large and medium cages are stacked in an ABAB layer fashion (Fig. 1F), the diffusion of substrates and intermediates has a very low barrier and short path, which facilitates the conversion of substrates. The catalytic performance of PCN-888-enR was also studied. It demonstrated a much worse catalytic performance than PCN-888-en, as displayed in Table 1. This can be attributed to the much lower GOx loading in the material and larger diffusion barrier between GOx and HRP that are not adjacent to each other.

**Table 1:** Kinetic parameters of GOx-HRP in free states, PCN-888-en and PCN-888-enR in the tandem reaction

	*K*_m_/mM	*V*_max_/mM s^–1^	*k*_cat_/s^–1^
Free	9.44	1.02 × 10^–3^	7.253 × 10^4^
PCN-888-en	9.67	1.96 × 10^–3^	2.411 × 10^4^
PCN-888-enR	11.71	1.50 × 10^–6^	7.946 × 10^1^

In comparison with many enzyme loaded silica materials, such as macroporous silica foams (MSF), immobilized enzymes always suffer activity loss, due to the partial protein unfolding or geometry disruption induced by the strong electrostatic interaction between the positively charged protein and negatively charged silica, however, PCN-888 is electrical neutral so the above mentioned issue no longer exists. For the enzyme loaded polymersomes, the encapsulated enzymes inevitably suffer from activity loss since organic solvents, such as tetrahydrofuran (THF), are always utilized in the enzyme encapsulation procedure. Moreover, the rate of reaction catalyzed by PCN-888-en is much faster than that of many polymersomes, in which the conversion of substrates can hardly reach a maximum rate within several hours, which is probably attributed to the higher enzyme encapsulation capacity of PCN-888 and the empty 2.0 nm cage of PCN-888 that greatly facilitate diffusion, in contrast to the poorly permeable polymersome membranes.^5^

The leaching of enzymes from PCN-888 was revealed to be negligible, indicating that PCN-888 can provide strong interactions between the cage and the immobilized moieties. As a result, the activity of PCN-888-en remained almost the same within four catalytic cycles, indicating its promising performance in multiple-cycle uses (ESI, Fig. S28†). In a previous report, Ma and coworkers have demonstrated the presence of π···π interactions between the immobilized enzyme and the conjugated ligands in a mesoporous cageMOF.^26^ The HTB ligand in PCN-888 can form a large conjugated system between the heptazine core and terminal benzene rings, which should lead to a strong interaction with the immobilized enzymes. At the same time, the small window size of the cages also physically prevents the leaching of the enzymes from the framework, although the enzyme has to somehow make its way into the cage, one possible way being unfolding and refolding.^26^ In contrast, enzyme leaching of mesoporous silica materials, such as SBA-15, has proved to be significant even after one cycle, resulting in a severe reduction of the catalytic activity.^9^

The stability of enzymes in a cellular environment is extremely important for the widespread biomedical applications of enzymatic nanoreactors.^1^ Digestion by a protease, such as trypsin, is a major deactivation pathway of enzymes *in vivo*.^28^ PCN-888-en has retained almost all of its activity after treatment with trypsin at 37 °C for 60 minutes, whereas the free enzymes lost two thirds of their activity after the same treatment. The protective effect of PCN-888 can be attributed to the small window openings of the enzyme-encapsulated cages, which makes the barrier of the immobilized enzymes approaching the active sites of trypsin extremely high.

## Conclusions

In summary, a novel hierarchical MOF, PCN-888, which possesses three types of mesoporous cages, is designed and synthesized for bi-enzyme coupling with a precise control of the enzyme distribution all over the material. The largest cage and the medium cage can only accommodate one molecule of GOx and HRP, respectively, indicating that a stepwise encapsulation procedure with a specific order (GOx first, then HRP) is a key operation to achieve the coupling. Control experiments demonstrate that bi-enzyme coupling failed to be established with a reversed encapsulation order. The high catalytic efficiency of the PCN-888 nanoreactor, good cycling performance as well as the protective effect of PCN-888 on the immobilized enzymes against trypsin digestion indicates that cageMOF tandem nanoreactors have the potential to be applied in more complex systems.

## Experimental

### Synthesis of PCN-888

AlCl_3_·6H_2_O (20 mg), HTB (10 mg) and TFA (0.1 mL) were dissolved in 2 mL DMF in a 4 mL pyrex vial. The mixture was kept in a 135 °C oven for 24 hours. The solid was collected by centrifuge. Yield: 4 mg.

### Enzyme immobilization of PCN-888

12 mg glucose oxidase (GOx) was dissolved in 2 mL water. 20 mg horseradish peroxidase (HRP) was dissolved in 2 mL water. 4 mg as-synthesized PCN-888 was washed with water twice and dispersed in 1 mL water. 1 mL GOx solution was added to the MOF slurry and incubated at room temperature for 50 minutes. The solid was collected by centrifuge and washed with DI water twice. The MOF was dispersed in 1 mL water. 1 mL HRP solution was added to the MOF slurry and incubated at room temperature for another 50 minutes. The solid was collected by centrifuge and washed with water twice. The supernatants were collected for the determination of the amount of immobilized enzymes in PCN-888. The uptake amount was determined by the bicinchoninic acid (BCA) method.^29^

### Activation of PCN-888

Freshly prepared PCN-888 was washed with DMF three times. The sample was evacuated with supercritical CO_2_ in a Tousimis Samdri PVT-3D critical point dryer. Briefly, the DMF-containing sample was placed in the chamber and DMF was completely exchanged with liquid CO_2_. After that the chamber containing the sample and liquid CO_2_ was heated up to around 40 °C and kept under a supercritical condition (typically 1300 psi) for 30 minutes. The CO_2_ was slowly vented from the chamber at around 40 °C, yielding a porous material. The yellow solid was further activated by heating at 150 °C for two hours.
