# Supplementary material for: Coupling two enzymes into a tandem nanoreactor utilizing a hierarchically structured MOF
Source: Chem Sci. 2016 Jul 14;7(12):6969–73. doi: 10.1039/c6sc01438k (PMC5356029; doi:10.1039/c6sc01438k)
Supplement: Supplementary file 1 [file SC-007-C6SC01438K-s001.pdf]

Supporting information

Coupling Two Enzymes into a Tandem Nanoreactor Utilizing a Hierarchically Structured MOF

Xizhen Lian[†], Ying-Pin Chen[†,‡], Tian-Fu Liu[†], Hong-Cai Zhou*[,†,‡]

[†]Department of Chemistry, Texas A&M University, College Station, Texas 77842-3012, United States

[‡] Department of Material Science and Engineering, Texas A&M University, College Station, Texas 77843, United States

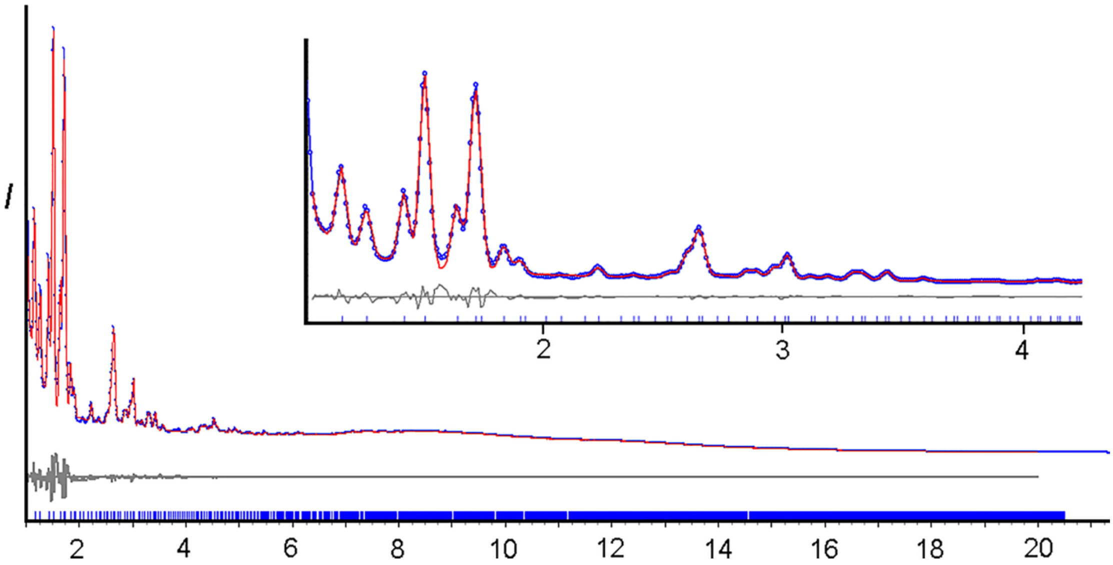

Figure S1. Rietveld refinement patterns of PCN-888 using synchrotron PXRD data (λ = 0.72768Å): observed (blue), calculated (red), and difference (grey) profiles are shown; the tick marks below the curves indicate Bragg positions. The X-ray diffraction pattern between 1.1° and 4.2° is magnified in the inset. The full pattern shows a precise match between the experimental PXRD data and those simulated from the proposed structure.

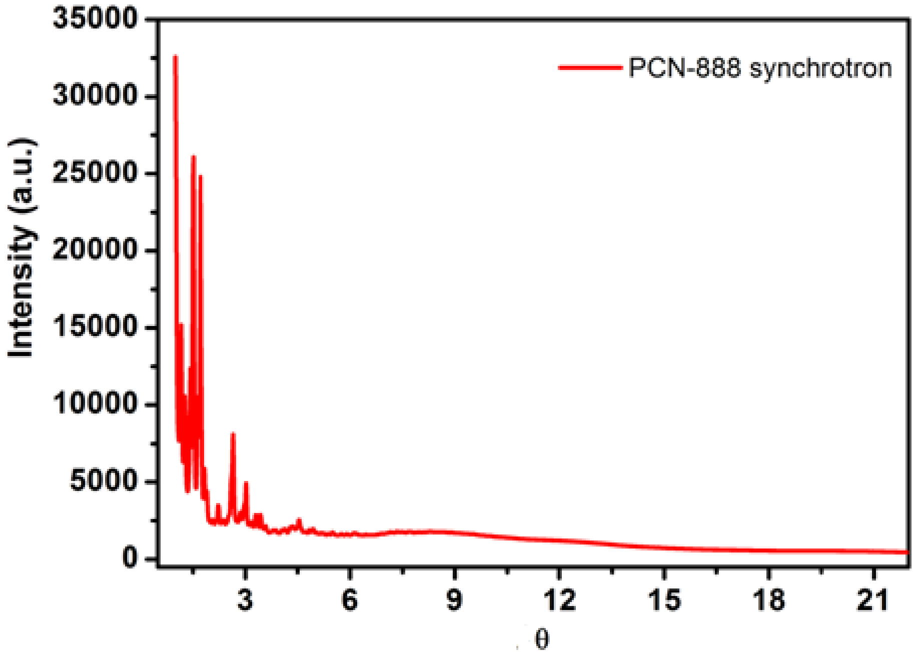

Figure S2. Powder X-ray diffraction of pristine PCN-888 from a Synchrotron source (λ = 0.72768 Å)

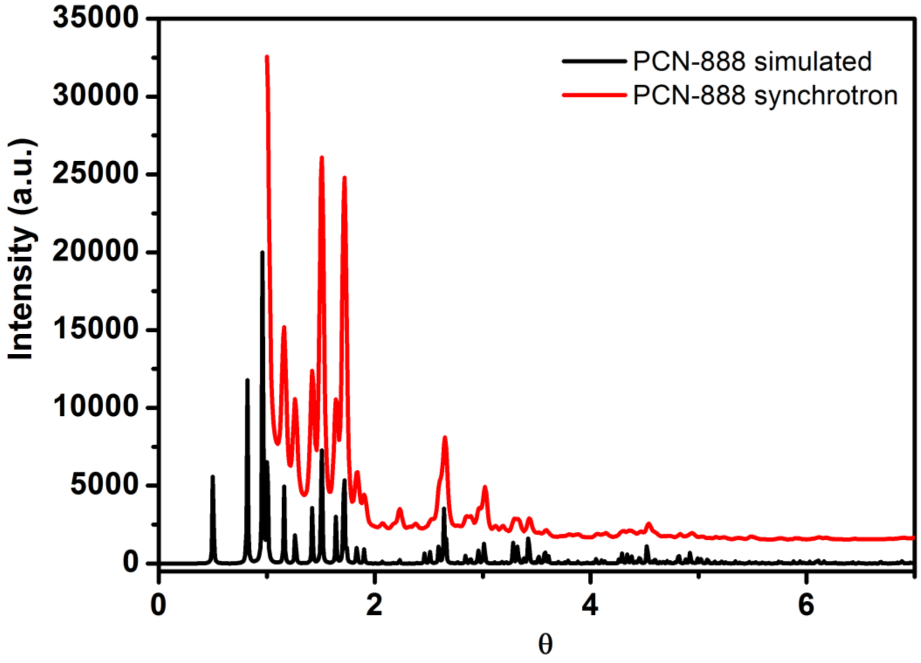

Figure S3. Comparison of experimental and simulated powder X-ray diffraction pattern of PCN-888.

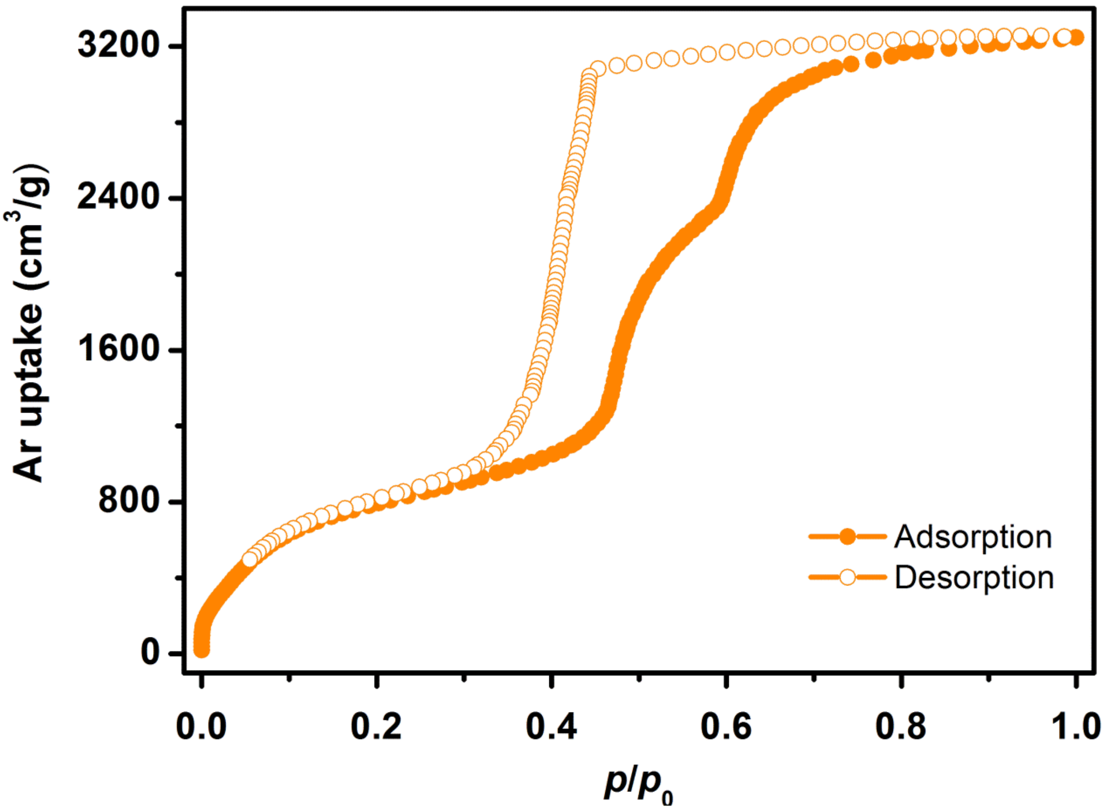

Figure S4. Ar isotherms of PCN-888 measured at 87 K.

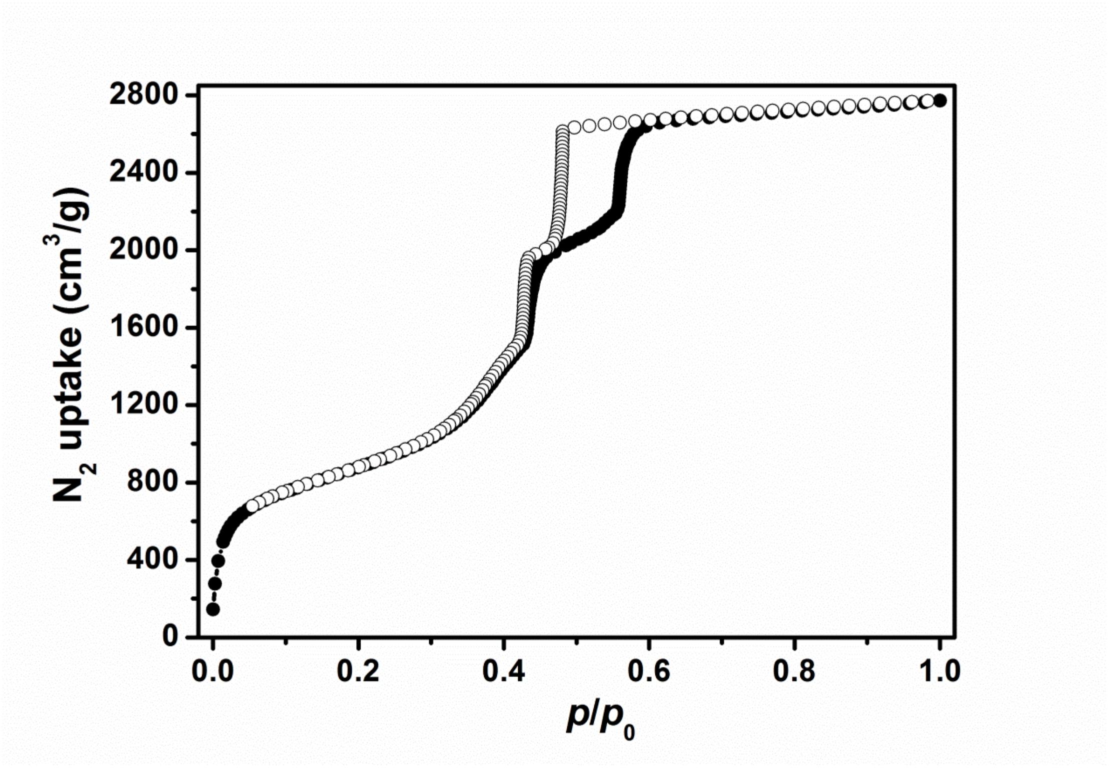

Figure S5. N_2 adsorption of pristine PCN-888 at 77K.

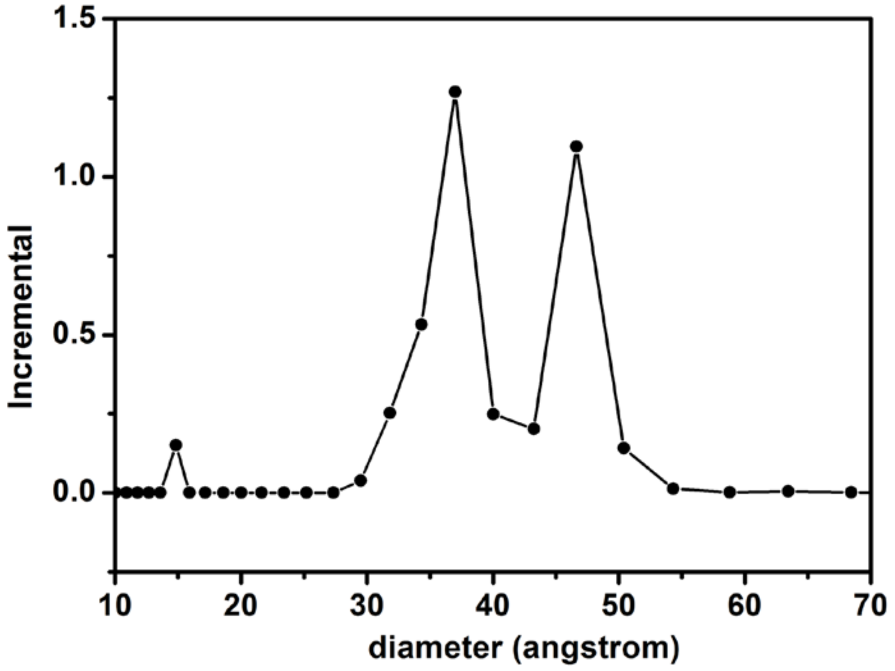

Figure S6. DFT pore size distribution of PCN-888 obtained from N_2 isotherm measured at 77K.

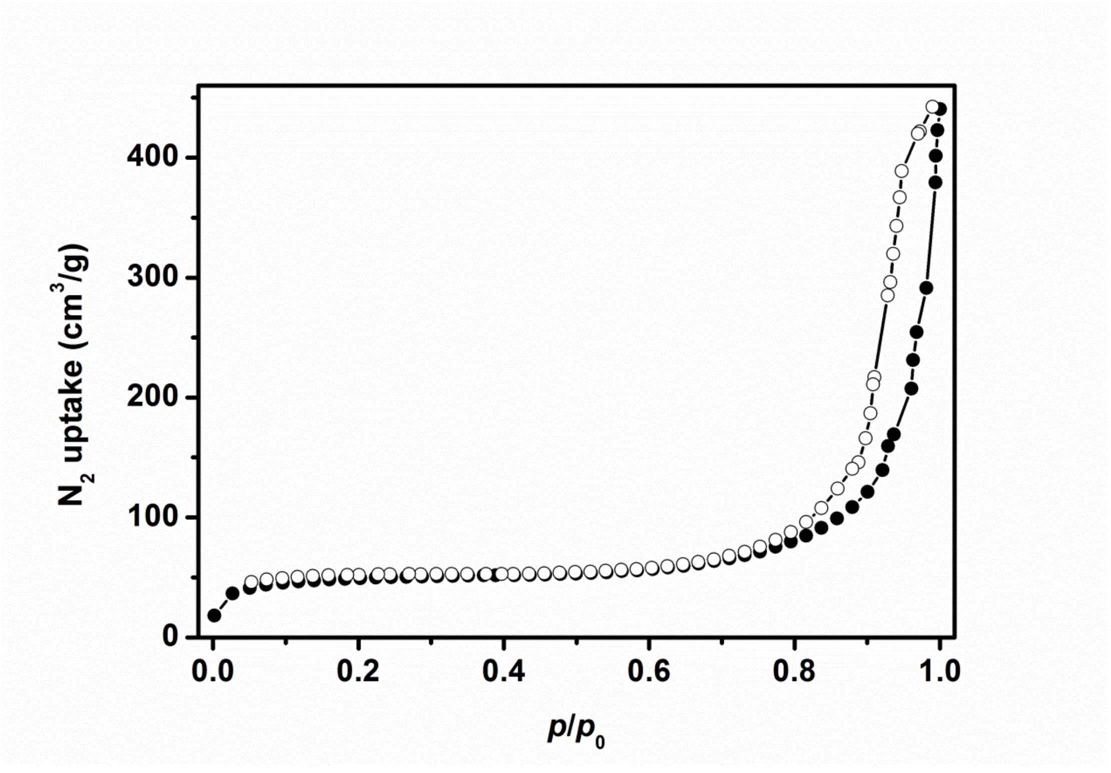

Figure S7. N_2 adsorption of PCN-888-en at 77K.

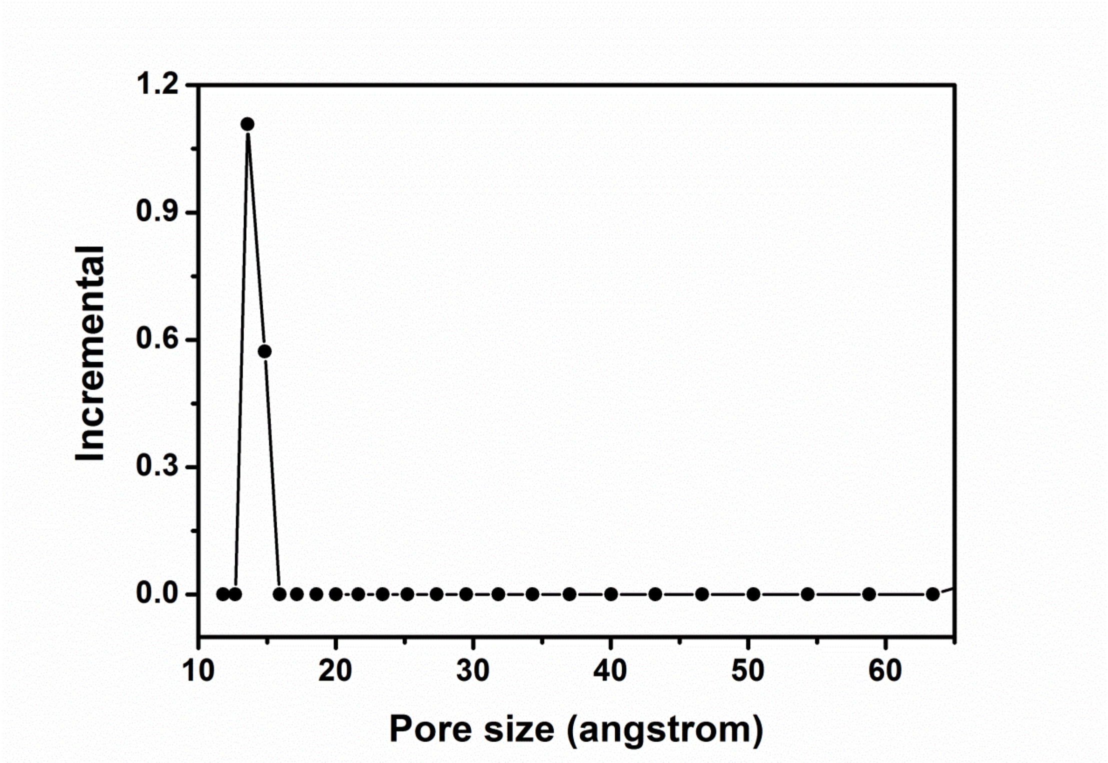

Figure S8. DFT pore size distribution of PCN-888-en obtained from N_2 isotherm at 77K.

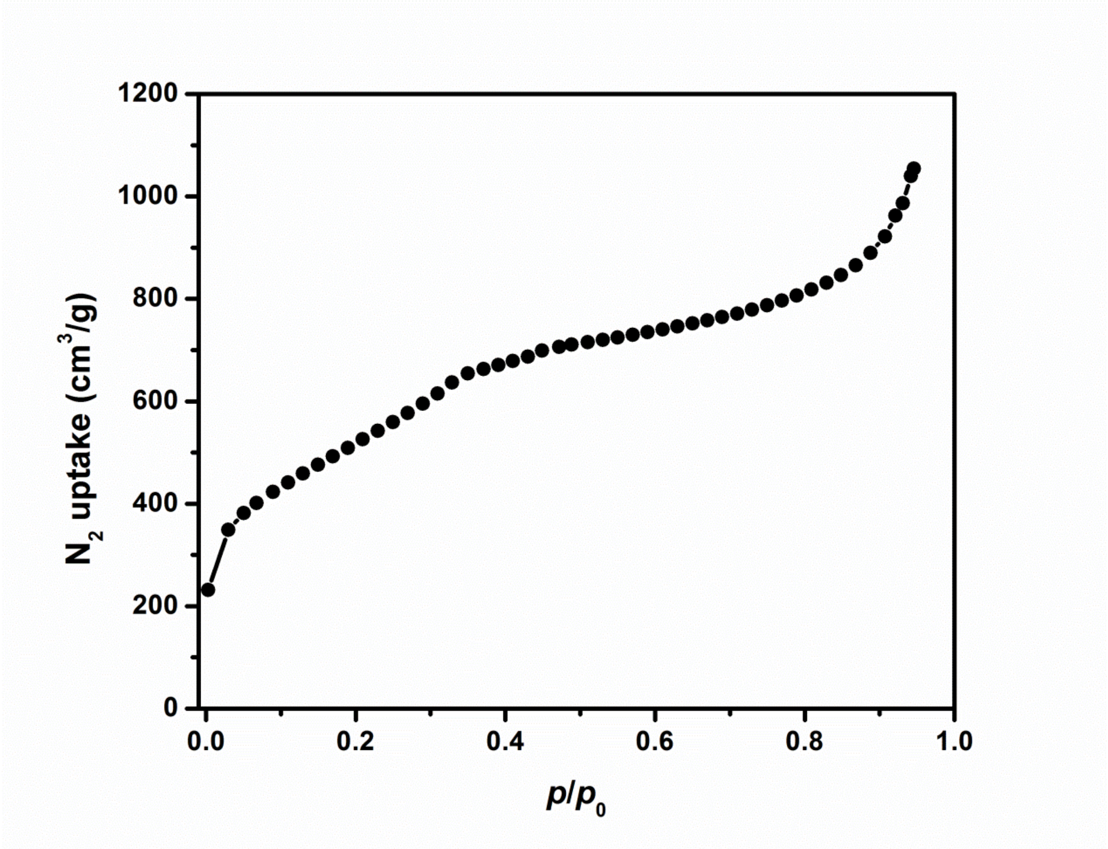

Figure S9. N_2 adsorption of GOx@PCN-888 at 77K.

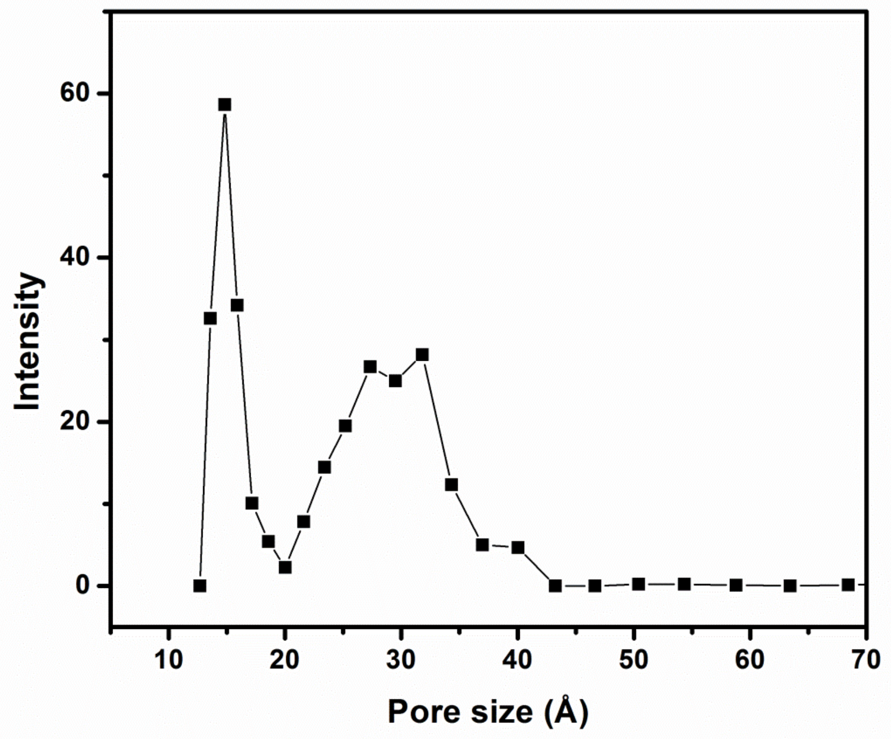

Figure S10. Pore size distribution of GOx@PCN-888 obtained from nitrogen isotherm at 77K.

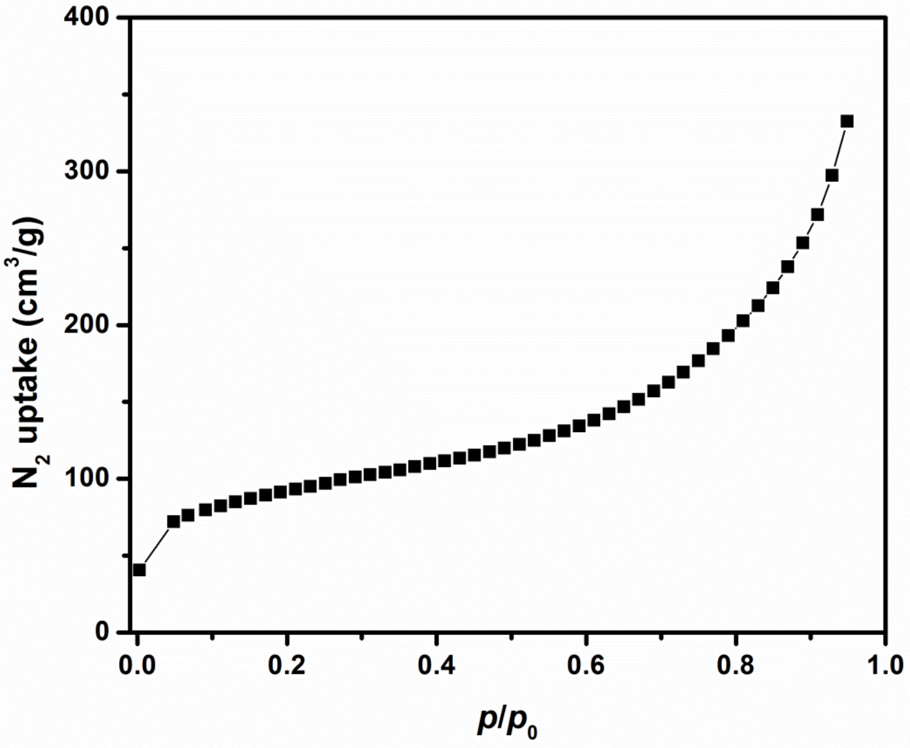

Figure S11. N2 isotherm of HRP@PCN-888 at 77K.

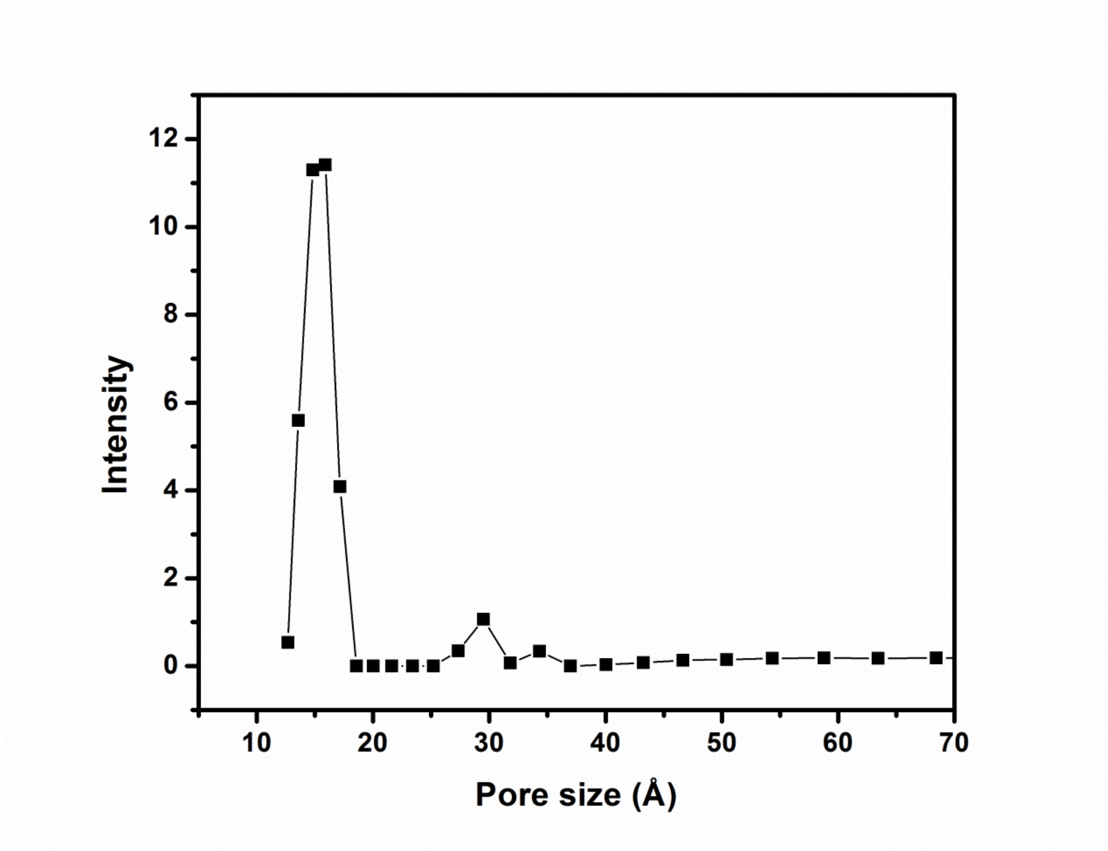

Figure S12. Pore size distribution of HRP@PCN-888 obtained from nitrogen isotherm at 77K.

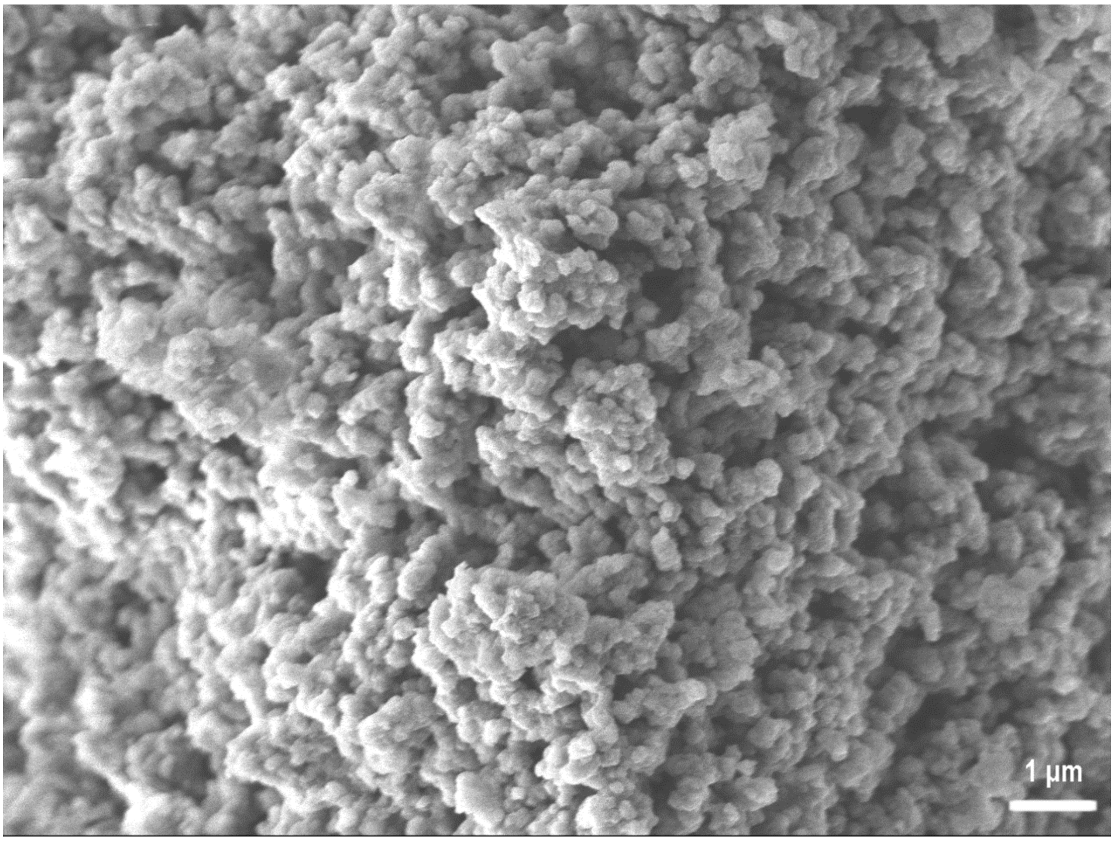

Figure S12. SEM images of pristine PCN-888.

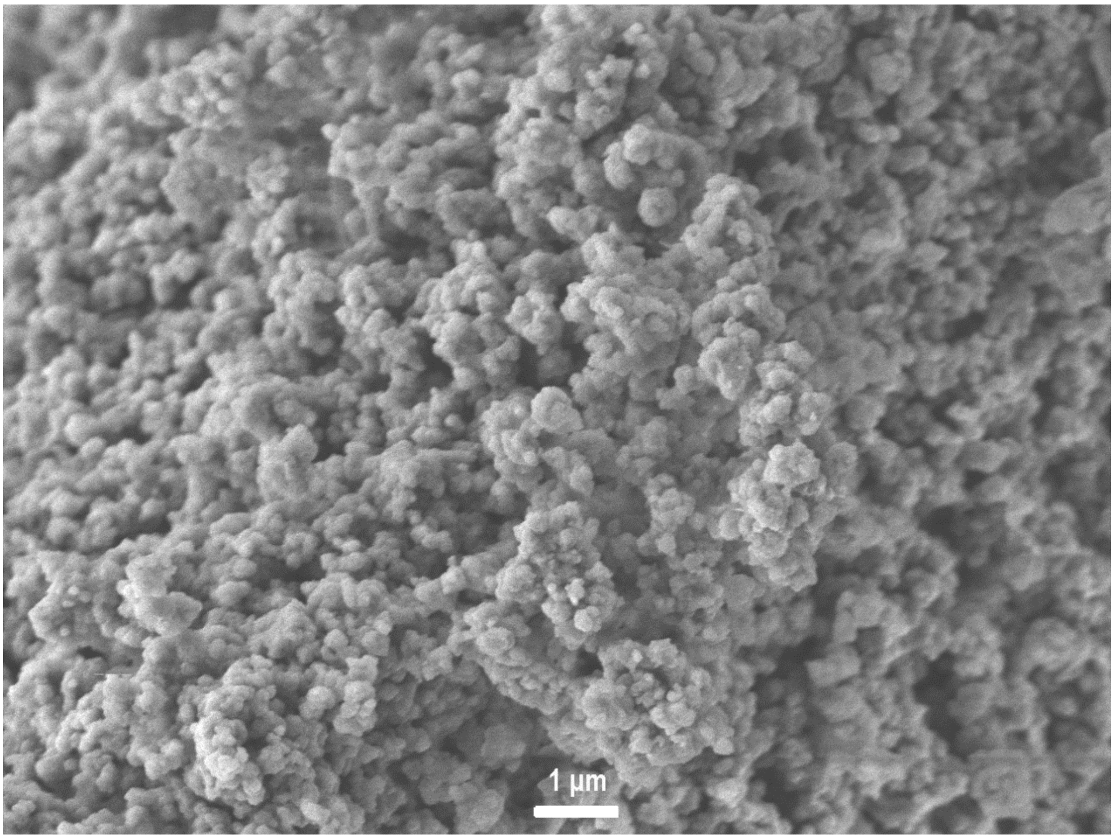

Figure S13. SEM image of GOx@PCN-888.

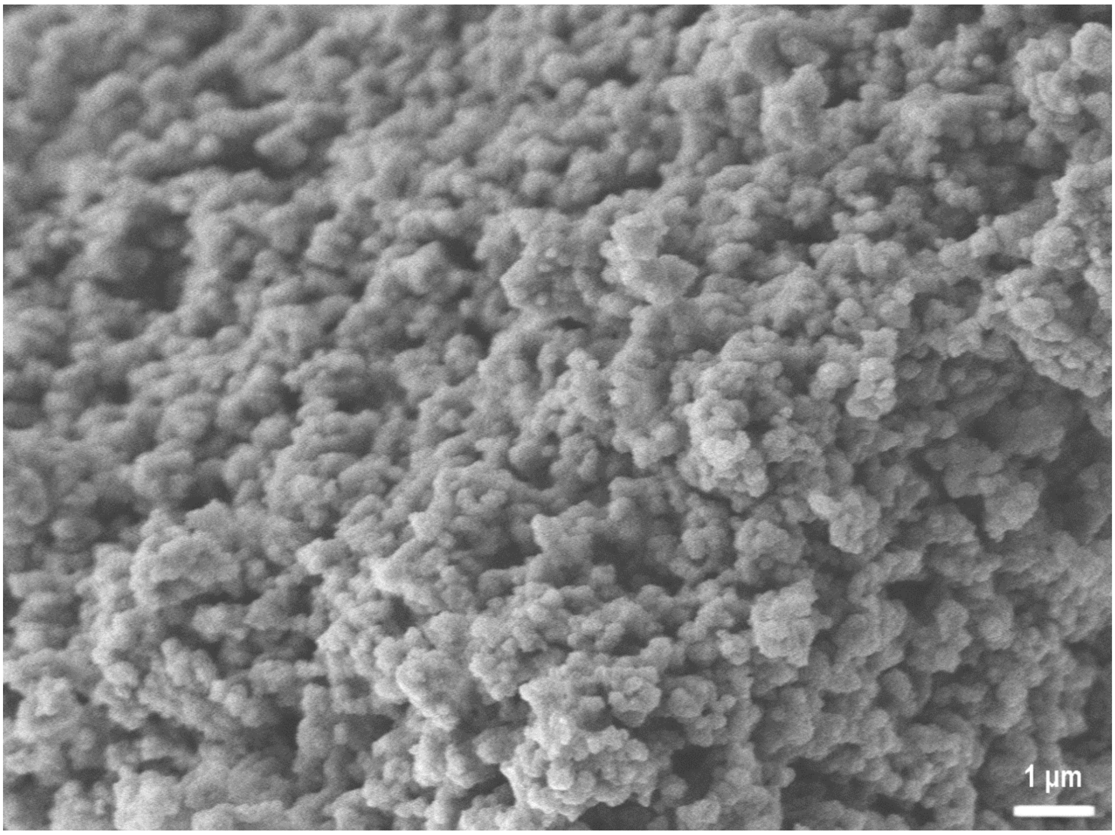

Figure S14. SEM image of HRP@PCN-888.

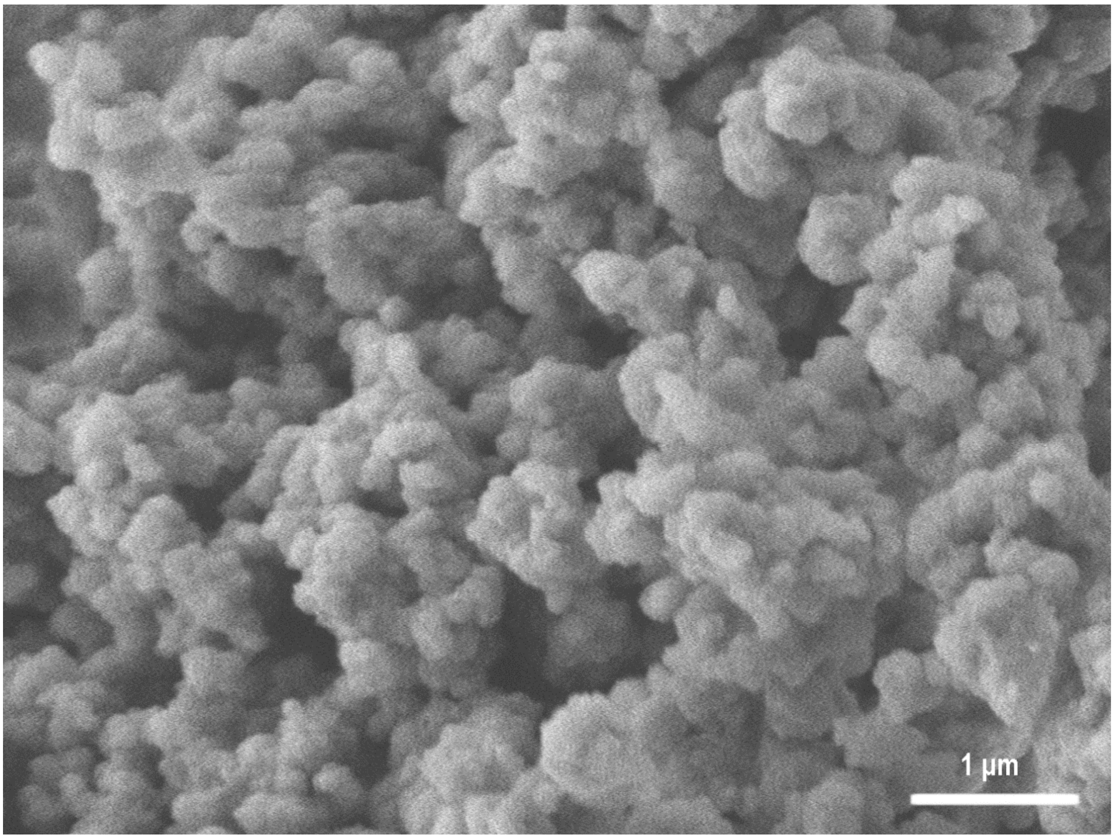

Figure S15. SEM image of PCN-888-en.

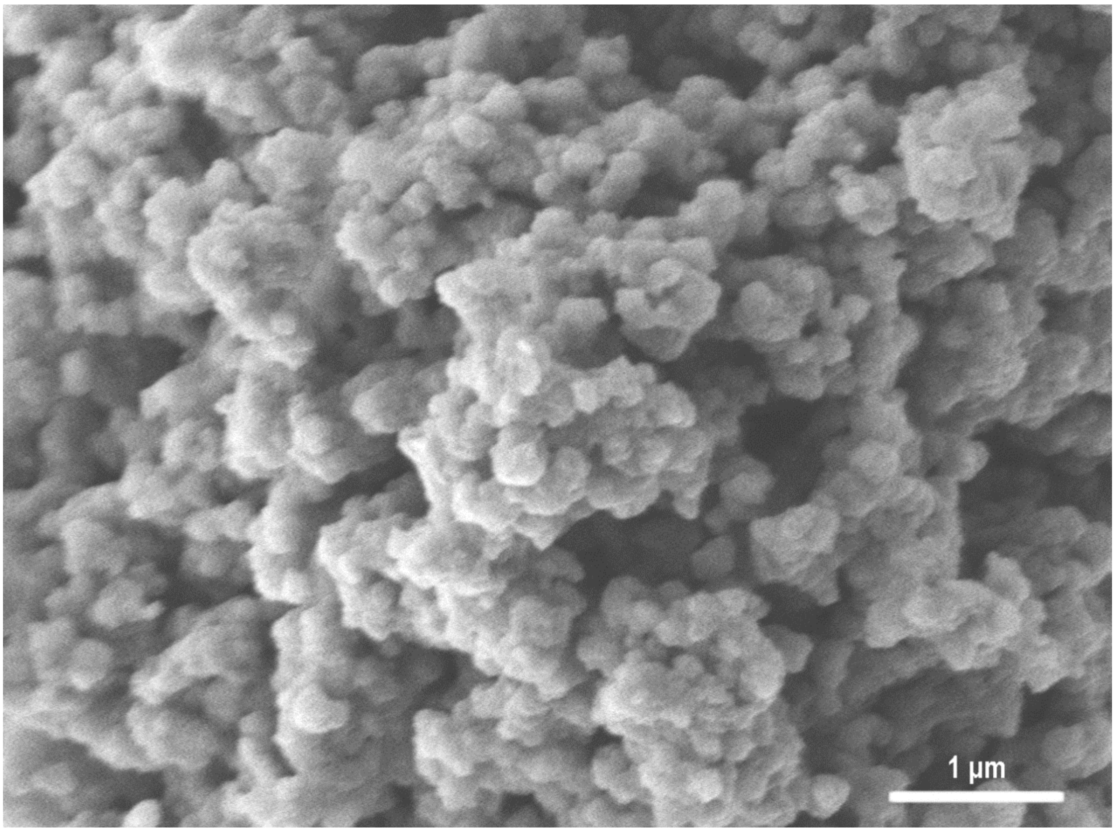

Figure S16. SEM image of PCN-888-en after 4-time catalysis cycle.

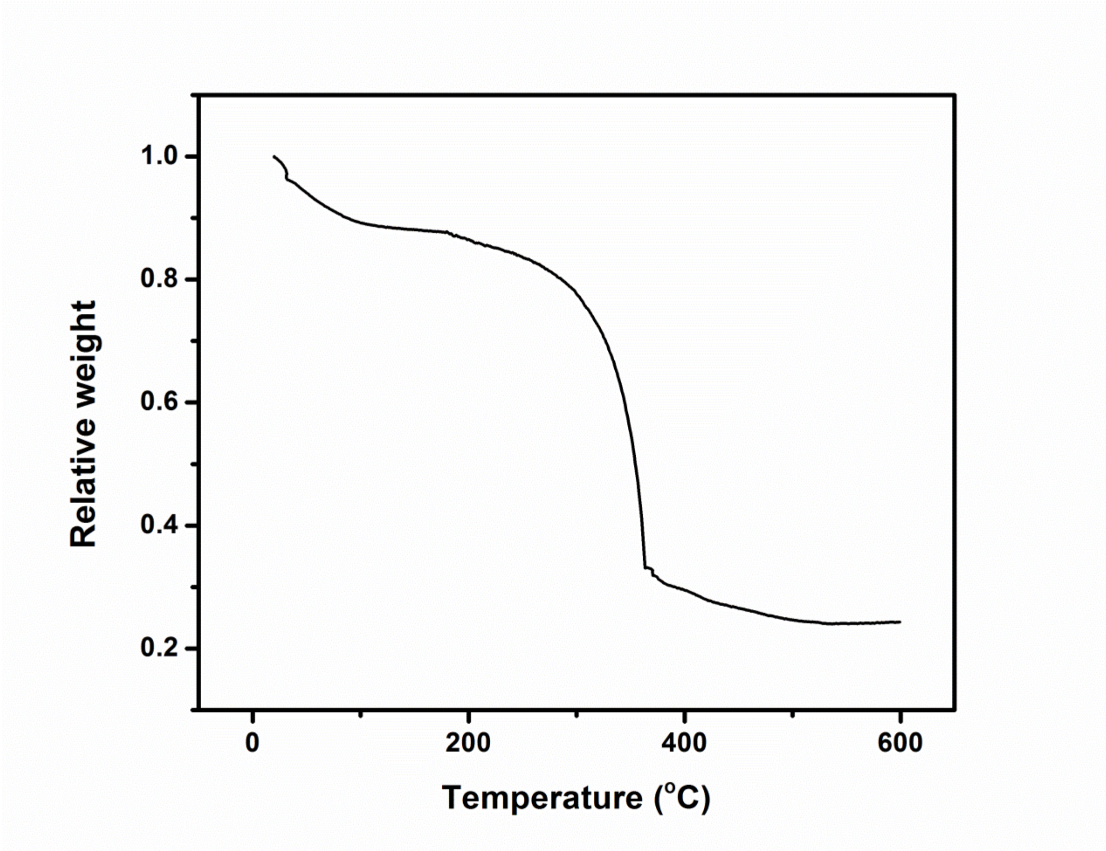

Figure S17. TG analysis of pristine PCN-888.

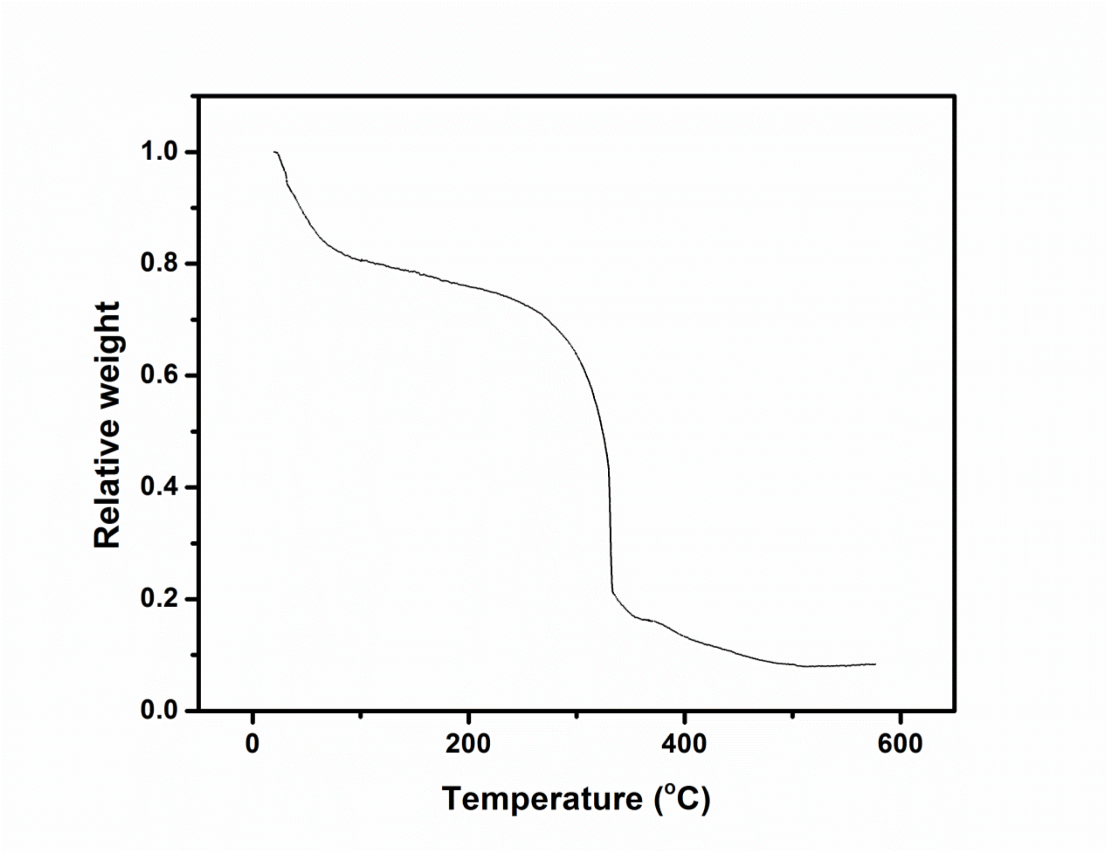

Figure S18. TG analysis of PCN-888-en.

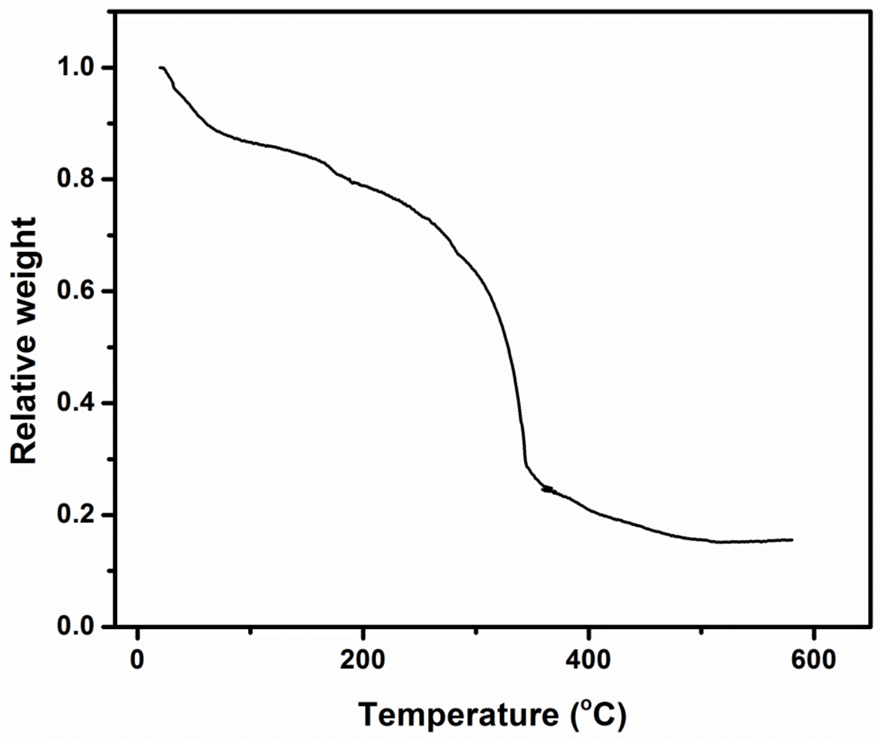

Figure S19. TG analysis of GOx@PCN-888.

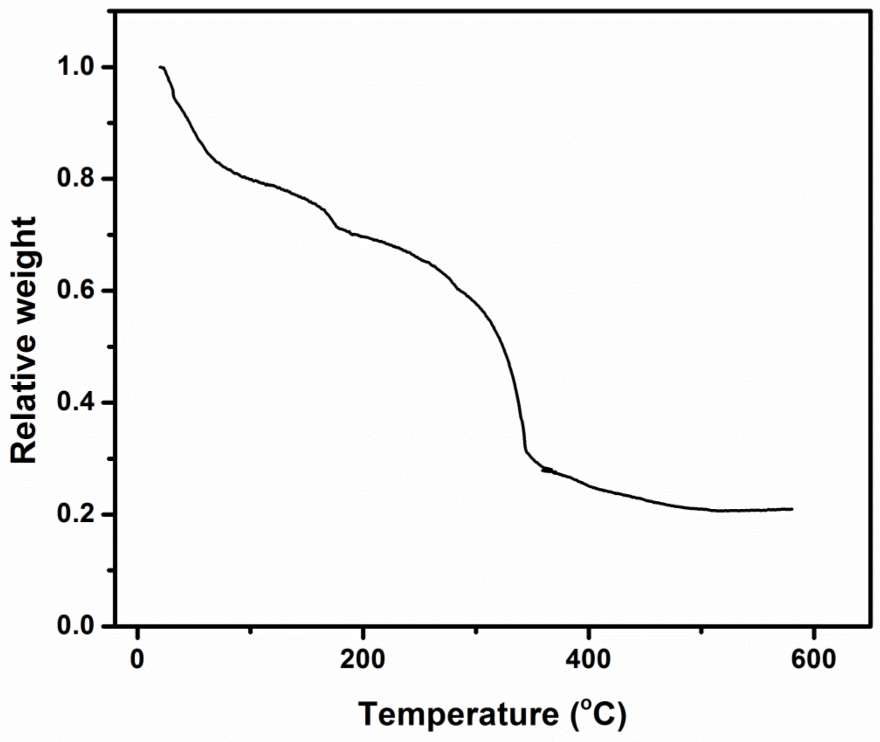

Figure S20. TG analysis of HRP@PCN-888.

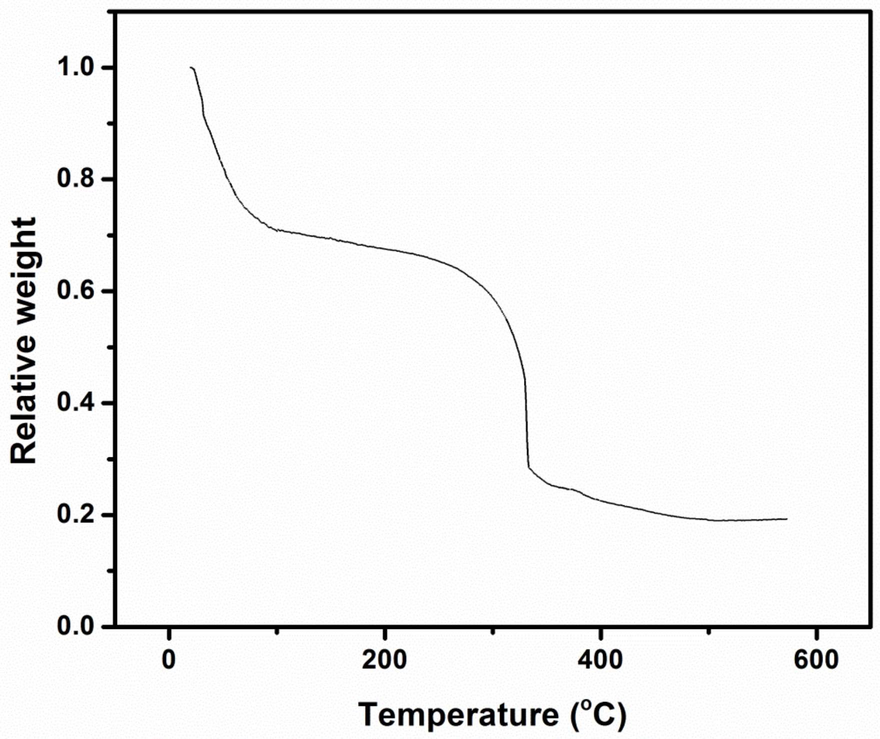

Figure S21. TG analysis of PCN-888-en after 4-time catalytic cycle.

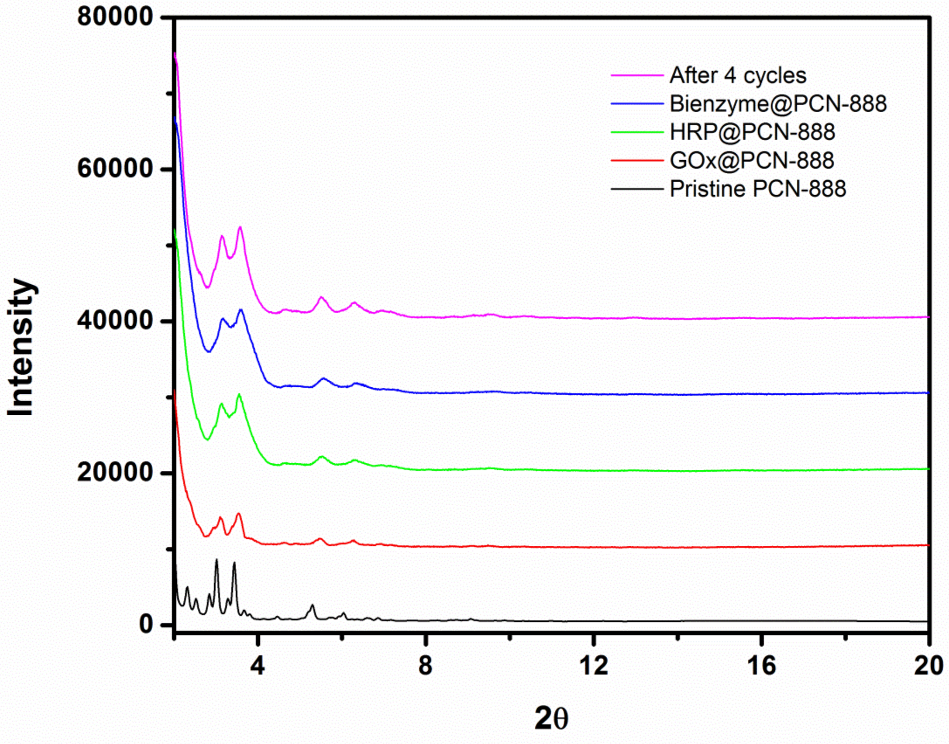

Figure S22. PXRD patterns of pristine PCN-888, GOx@PCN-888, HRP@PCN-888, PCN-888-en

(bienzyme incorporated) and PCN-888-en after a 4-time catalytic cycle.

Materials and Instruments

Aluminum chloride hexahydrate ($AlCl_3 \cdot 6H_2O$), anhydrous aluminum chloride, chromium(VI) oxide (CrO_3), 2,2'-azino-bis(3-ethylbenzothiazoline-6-sulphonic acid) diammonium salt (ABTS) and N,N-diethylformamide (DEF) is purchased from Alfa Aesar. Phosphorous pentachloride (PCl_5), phosphorous oxochloride ($POCl_3$), glucose and horseradish peroxidase (HRP) is purchased from Sigma Aldrich. Glucose oxidase and trypsin is purchased from MP biomedicals.

Synchrotron powder X-ray diffraction (PXRD) was carried out on a Bruker D8-Discover diffractometer equipped with a Mo sealed tube (λ = 0.72768Å) on the beamline 17-BM at the Advanced Photon Source, Argonne National Laboratory. Nuclear magnetic resonance (NMR) data were collected on a Mercury 300 spectrometer. Low pressure gas adsorption measurements were performed on an ASAP 2020 with the extra-pure quality gases. The UV-Vis absorption spectra were recorded on a Shimadzu UV-2450 spectrophotometer.

Scheme S1. Isoreticular extension strategy and synthetic routes of HTB ligand.

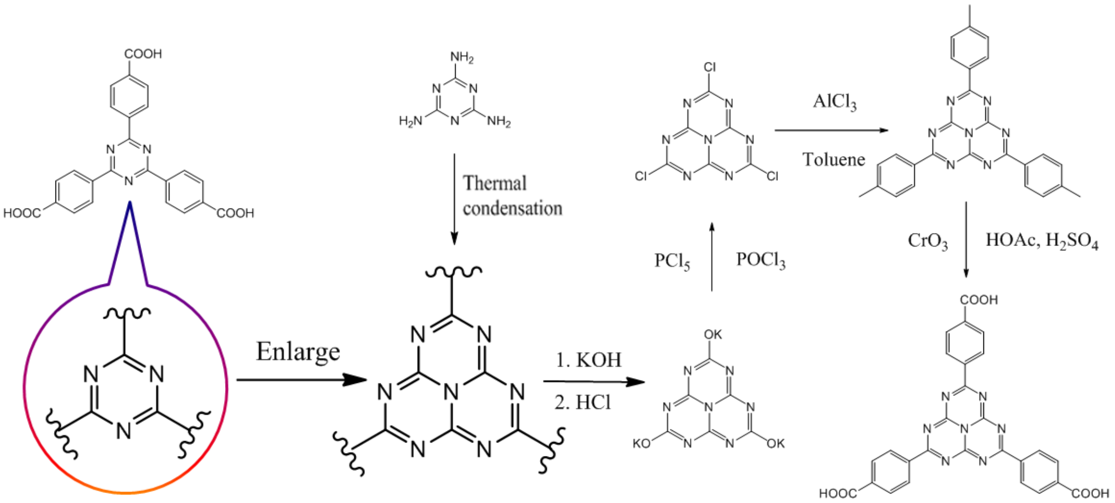

Synthesis of HTB

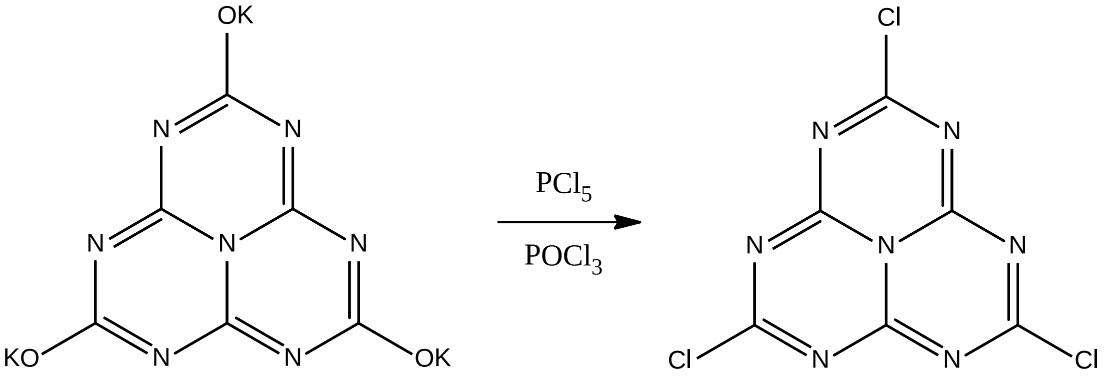

1 **2**

3.87 g **1** and 8.64 g PCl_5 was charged in a Schlenk bottle. The mixture was heated at 130^oC under vacuum overnight. 70 mL $POCl_3$ was added to the solid mixture and refluxed for 6 hours. The color of the reaction slurry turned from white to yellow. The solvent was

removed and the residue solid was washed with cold water. Yield: 3.2 g.

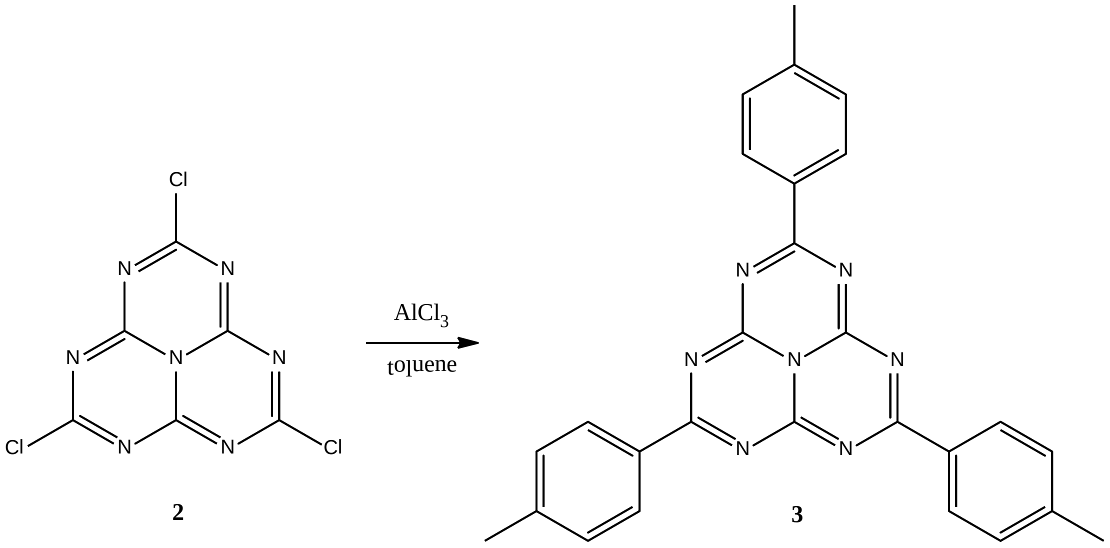

In a 100 mL Schlenk flask was charged 8 g anhydrous $AlCl_3$ and 20 mL anhydrous toluene. The mixture was heated to 60 ^oC under stirring. 3.2 g compound **2** was added to the flask in small portions over 20 minutes. The reaction mixture changed from yellow-green to dark red once compound **2** was added in and white gas was generated. The reaction was kept at 60 ^oC for 5 hours and it was cooled down to room temperature. The mixture was poured into ice water with rigorous stirring. The resulting yellow solid was filtered and washed with water and cold THF. Yield: 1.5 g. ^1H NMR (300 Hz, Benzene), 2.017 (s, 3H), 7.024 (d, 2H), 8.854 (d, 2H).

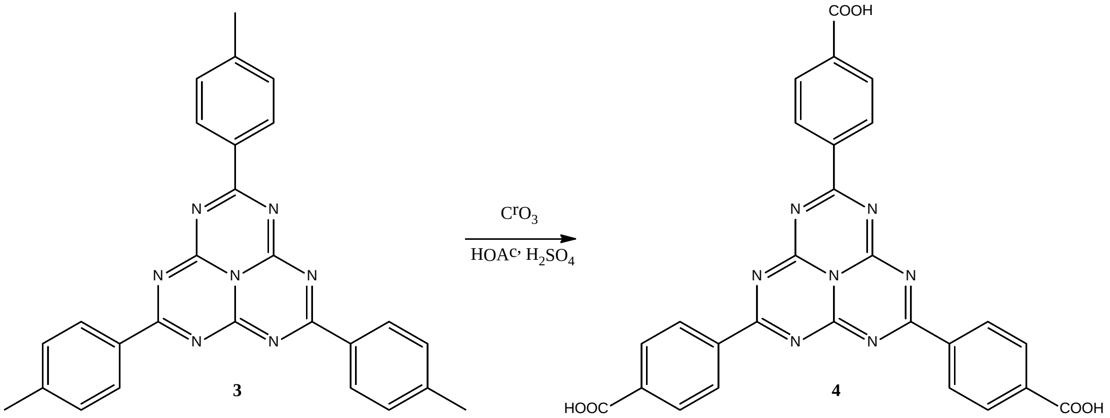

1 g compound **3**, 20.5 g HOAc, 1.23 mL H_2SO_4 was added in a 100 mL flask in ice bath. 2.03 g CrO_3 was added slowly to the flask. Then 1.35 g Ac_2O was added in. The flask was kept at 0^oC for 10 minutes and it was allowed to stay at room temperature overnight. The reaction mixture was poured into 300 mL deionized water and stirred for 1 hour. The solid was collected by centrifuge and washed with water for three times. The product was dissolved with 2.5 M KOH aqueous solution and the residue solid was removed by centrifuge. To the clear solution was added 6 M HCl until pH=1. The product is collected by filtration. Yield: 0.5 g, yellow solid. ^1H NMR (300MHz, DMSO): 8.14 (d, 2H), 8.50 (d, 2H).

Synthesis of PCN-888

$AlCl_3{\cdot}6H_2O$ (20 mg), HTB (10 mg) and TFA (0.1 mL) was dissolved in 2 mL DEF in a 4 mL pyrex vial. The mixture was kept in a 135ºC oven for 10 hours. The solid was collected by centrifuge. Yield: 4 mg.

Enzyme immobilization of PCN-888

12 mg glucose oxidase (GOx) was dissolved in 2 mL water. 20 mg horseradish peroxidase (HRP) was dissolved in 2 mL water. 4 mg as-synthesized PCN-888 was washed with water twice and dispersed in 1 mL water. 1 mL GOx solution was added to the MOF slurry and incubated at room temperature for 50 minutes. The solid was collected by centrifuge and washed with DI water twice. The MOF was dispersed in 1 mL water. 1mL HRP solution was added to the MOF slurry and incubated at room temperature for another 50 minutes. The solid was collected by centrifuge and washed with water twice. The supernatants were collected for the determination of the amount of immobilized enzymes in PCN-888.

Activation of PCN-888

Freshly prepared PCN-888 was washed with DMF for three times. The sample was evacuated with supercritical CO_2 in a Tousimis Samdri PVT-3D critical point dryer. Briefly, the DMF-containing sample was placed in the chamber and DMF was completely exchanged with liquid CO_2. After that the chamber containing the sample and liquid CO_2 was heated up around 40 °C and kept under the supercritical condition (typically 1300 psi) for 30 minutes. The CO_2 was slowly vented from the chamber at around 40 °C, yielding porous material. The yellow solid was further activated by heating at 150°C for two hours.

The measurement of kinetic parameters of PCN-888-en/PCN-888-enR

ABTS and glucose are dissolved in 1mL 50 mM pH=7.4 tris-HCl buffer solution to yield desired concentration of ABTS (10 mM) and glucose (0.6-9 mM) solution. MOF powder is added in the solution and the system is monitored by UV-vis spectrometer at 403 nm spontaneously. The measurement is stopped when the adsorption reaches plateau.

The measurement of kinetic parameters of free enzymes

The procedure is similar with that of MOF nanoreactors instead of the glucose concentration range (1-9 mM), GOx concentration (0.0141 μM,) and HRP concentration (0.05057 μM).

Trypsin digestion conditions of PCN-888-en

PCN-888-en (10 mg) was suspended in 2 mL 50 mM pH=7.4 tris-HCl buffer. 2 mg trypsin was added and the media was incubated at 37 ºC for 60 minutes. The powder was obtained by centrifugation and washed with fresh buffer for 3 times.

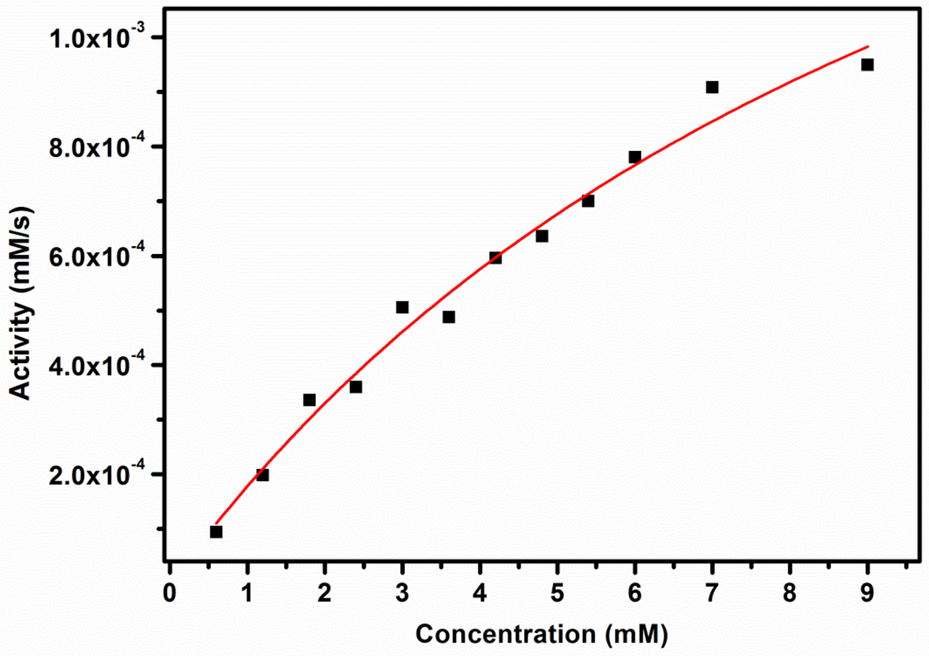

Figure S23. The Michaelis-Menten plot for the effect of increasing concentration of glucose on the initial velocity of PCN-888-en nanoreactor.

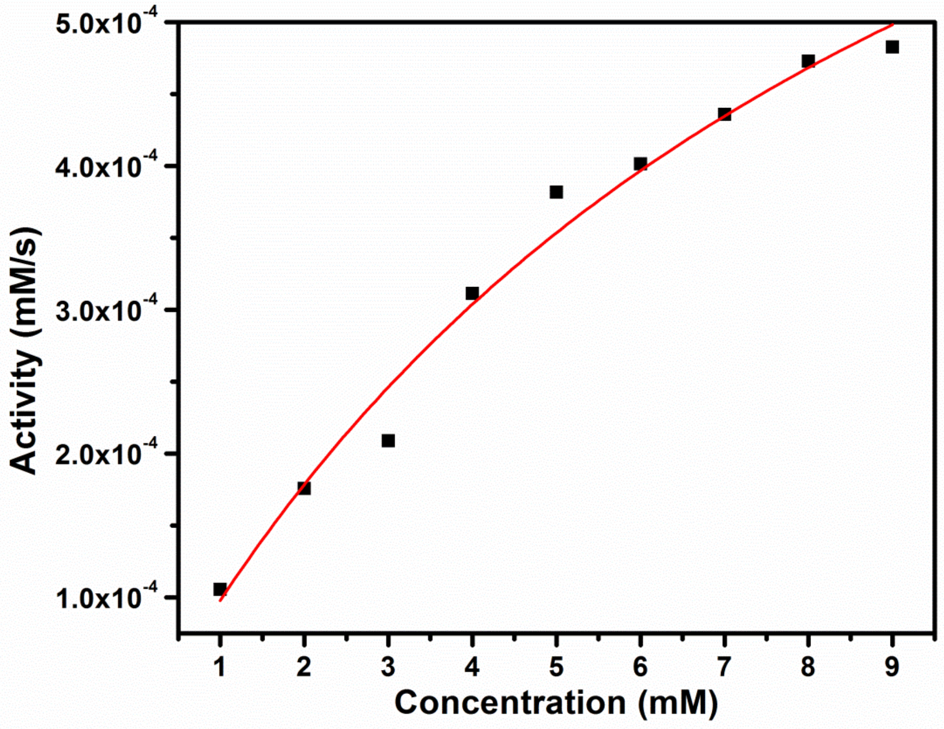

Figure S24. The Michaelis-Menten plot for the effect of increasing concentration of glucose on the initial velocity of free GOx and HRP enzymes.

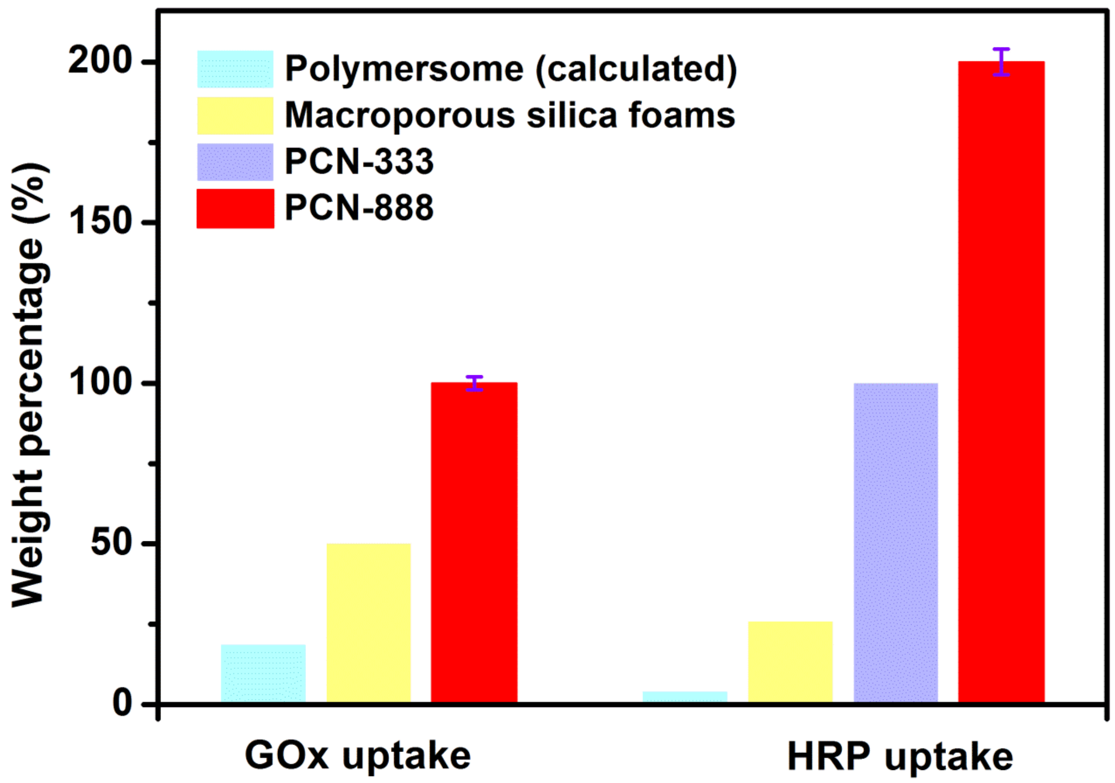

Figure S25. enzyme uptake capacity (GOx, HRP) of PCN-888 compared with other porous materials, such as polymersome (calculated value) (cyan); macroporous silica foams (MSF) (yellow); PCN-333 (blue).

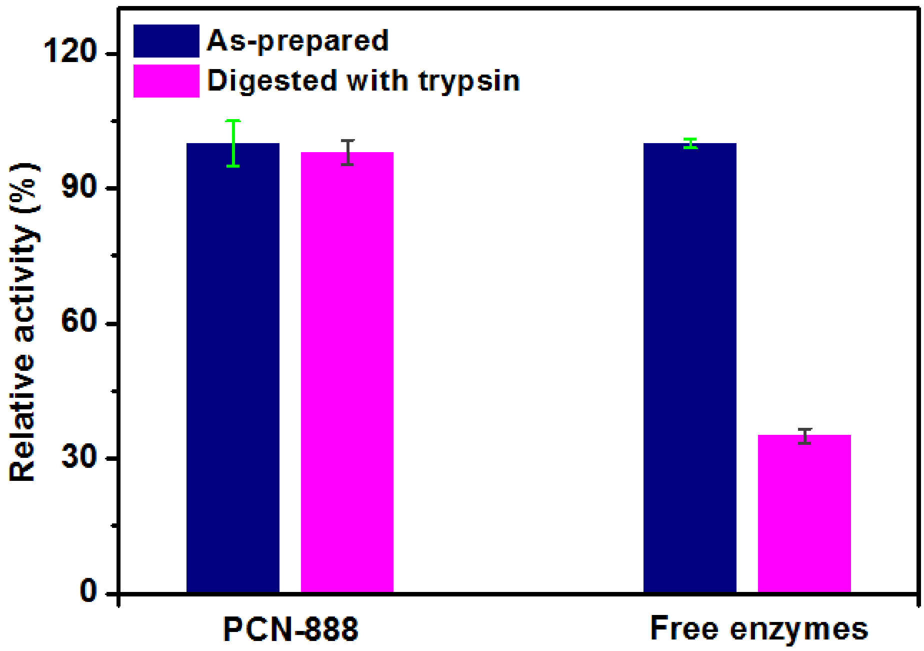

Figure S26. Stability of PCN-888 nanoreactor compared with free enzymes incubated in an aqueous solution containing 1 mg/mL trypsin at 37 ^{o}C for 1 hour.

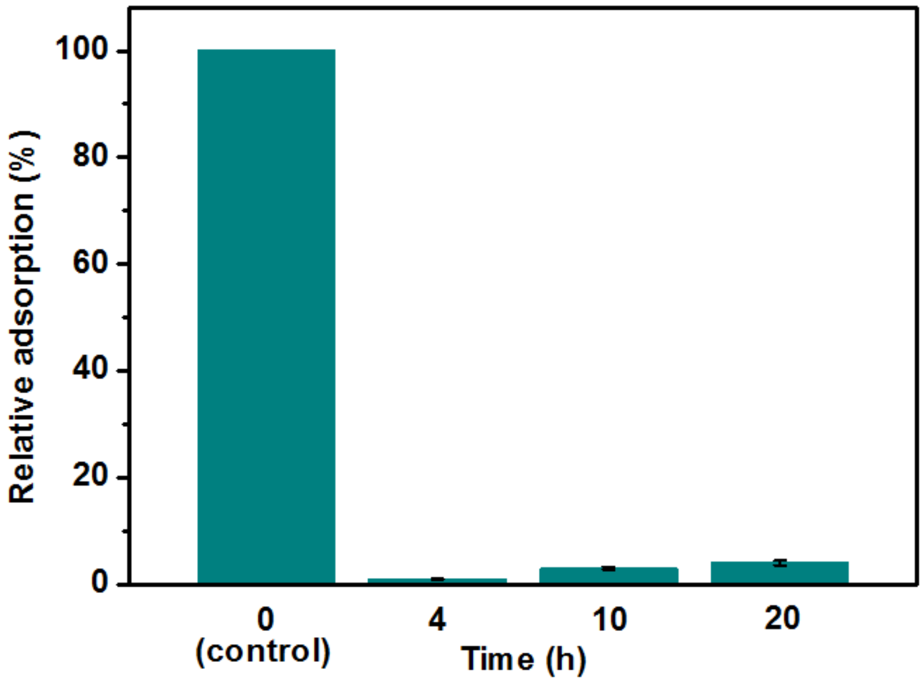

Figure S27. Leaching test of PCN-888 nanoreactor at 30, 60 and 120 minutes. The amount of leaching enzymes were determined by Thermo Scientific Pierce^{TM} BCA protein assay. The 100% standard solution at 0 minute was composed of 0.14 μM GOx and 1.1 μM HRP in water and room temperature.

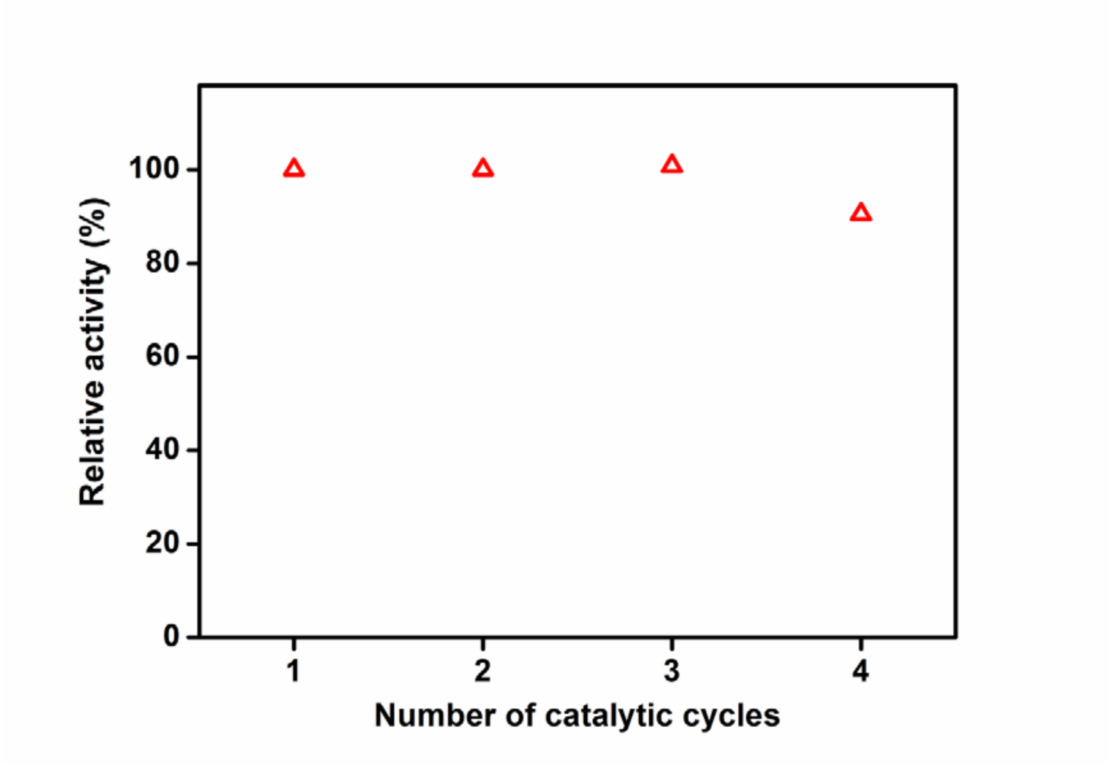

Figure S28. Reusability of PCN-888-en.

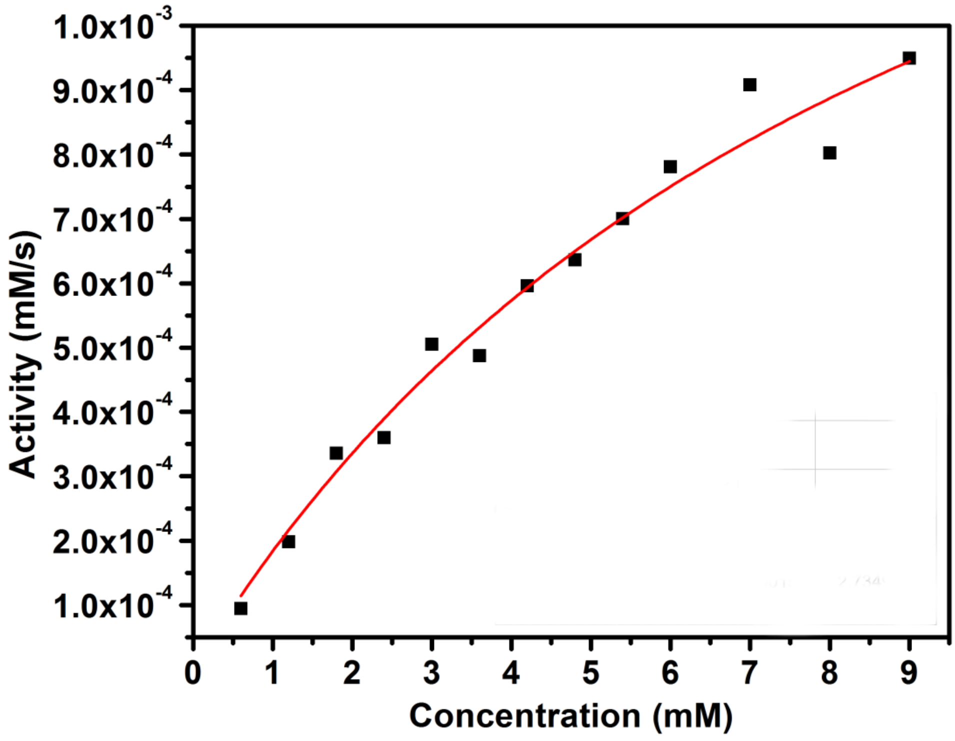

Figure S29. Catalytic activity of PCN-888-en after trypsin digestion.

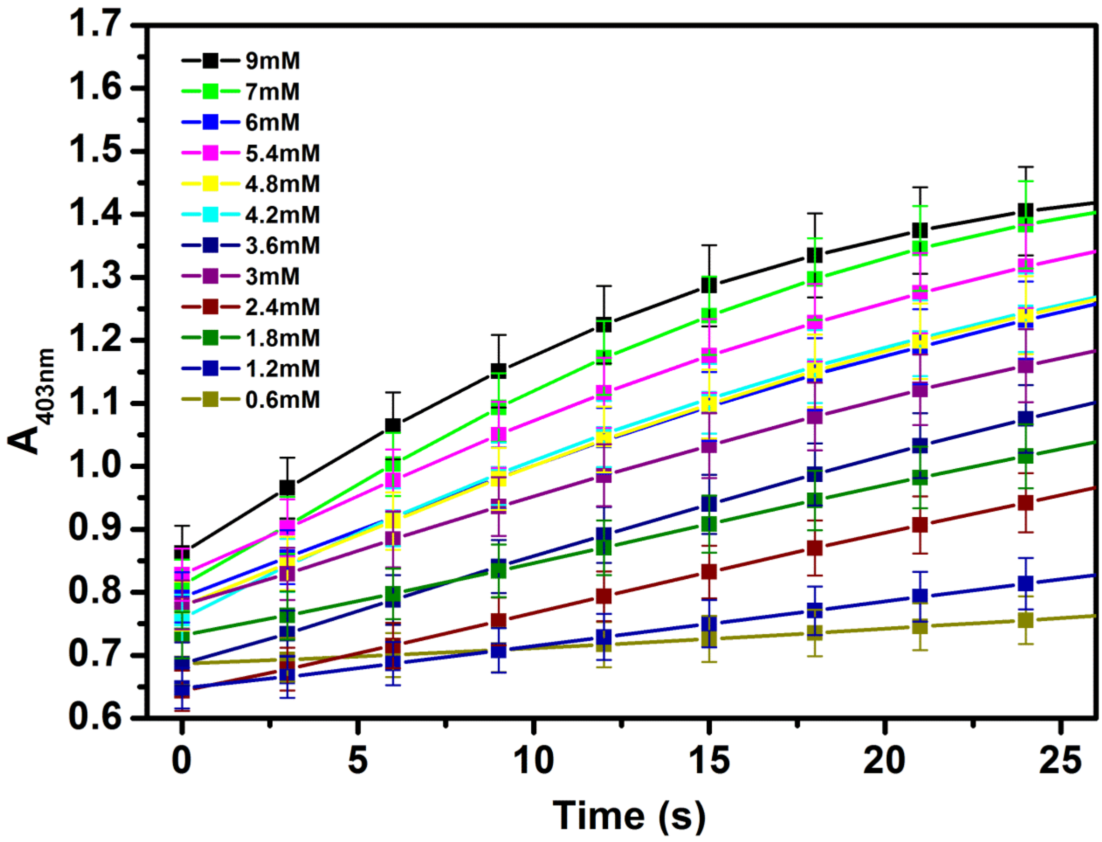

Figure S30. UV-vis data of PCN-888-en. All experiments are repeated for 3 times.

Table S1: Crystallographic data, experimental conditions for powder X-ray data collection and results of the Rietveld analysis of PCN-888(Al).

PCN-888(Al) CCDC	
Chemical formula	$C_{54}\ H_{24}\ N_{14}\ O_{16}\ Al_3$
Formula weight	1292.41
Crystal system	Cubic
Space group	$Fd\ \bar{3}m$
a/Å	143.574(9)
Temperature/K	295
Wavelength/Å	0.72768
2θ range/°	1.1~20.0
R_p	1.81
R_{wp}	3.49
R_{exp}	2.42
GOF	1.44

Refinement result of atomic coordinates in the PCN-888

H86	2	0.906260	0.760550	0.121150	1.000000	0
H87	2	0.903690	0.772160	0.109160	1.000000	0
H88	2	0.884600	0.786740	0.127270	1.000000	0
H89	2	0.887160	0.775070	0.139260	1.000000	0
H94	2	0.912340	0.784850	0.076020	1.000000	0
H95	2	0.924220	0.783750	0.064070	1.000000	0
H96	2	0.919940	0.812700	0.057220	1.000000	0
H97	2	0.908080	0.813840	0.069250	1.000000	0
H98	2	0.876740	0.823440	0.118840	1.000000	0
H99	2	0.871710	0.838830	0.123880	1.000000	0
H100	2	0.879960	0.850140	0.097280	1.000000	0
H101	2	0.885000	0.834820	0.092280	1.000000	0
C30	1	0.930970	0.797750	0.053140	1.000000	0
C31	1	0.923110	0.798150	0.059760	1.000000	0
C32	1	0.918260	0.806540	0.061200	1.000000	0
C33	1	0.911440	0.807200	0.068090	1.000000	0
C34	1	0.909320	0.799490	0.073670	1.000000	0
C35	1	0.913850	0.790980	0.071910	1.000000	0
C36	1	0.920680	0.790330	0.065060	1.000000	0
C37	1	0.903080	0.800380	0.081810	1.000000	0
C38	1	0.893820	0.809470	0.091880	1.000000	0
C39	1	0.897540	0.794000	0.095800	1.000000	0
C40	1	0.888760	0.803150	0.106180	1.000000	0
C41	1	0.893100	0.787870	0.110290	1.000000	0
C42	1	0.885220	0.818710	0.102410	1.000000	0
C43	1	0.893960	0.780410	0.117280	1.000000	0

C44	1	0.877400	0.829190	0.114030	1.000000	0
C45	1	0.881360	0.827890	0.105170	1.000000	0
C46	1	0.882030	0.835550	0.099160	1.000000	0
C47	1	0.879130	0.844360	0.102040	1.000000	0
C48	1	0.875470	0.845740	0.111020	1.000000	0
C49	1	0.873320	0.855280	0.114330	1.000000	0
C50	1	0.874490	0.838020	0.116920	1.000000	0
C67	1	0.898880	0.760170	0.138630	1.000000	0
C68	1	0.896970	0.766920	0.131160	1.000000	0
C69	1	0.901480	0.766190	0.122550	1.000000	0
C70	1	0.899960	0.772840	0.115660	1.000000	0
C71	1	0.890750	0.774320	0.132680	1.000000	0
C72	1	0.889280	0.781010	0.125820	1.000000	0
N9	3	0.889790	0.817710	0.094160	1.000000	0
N10	3	0.884760	0.811390	0.108390	1.000000	0
N11	3	0.893280	0.802180	0.097910	1.000000	0
N12	3	0.898770	0.808580	0.083870	1.000000	0
N13	3	0.902380	0.793140	0.087820	1.000000	0
N14	3	0.888710	0.796010	0.112360	1.000000	0
N15	3	0.897450	0.786930	0.101970	1.000000	0
O7	4	0.875300	0.861990	0.109000	1.000000	0
O5	4	0.869850	0.856380	0.122370	1.000000	0
O9	4	0.932450	0.804840	0.048160	1.000000	0
O14	4	0.935990	0.790480	0.052860	1.000000	0
O22	4	0.893650	0.760310	0.145720	1.000000	0
O21	4	0.905600	0.754530	0.137740	1.000000	0
O4	4	0.891420	0.891420	0.136560	0.500000	0
O6	4	0.875000	0.875000	0.123140	0.250000	0
Fe4	5	0.883300	0.883300	0.129910	0.500000	0
Fe5	5	0.875000	0.875000	0.109610	0.250000	0
O15	4	0.942960	0.774200	0.048300	1.000000	0
Fe2	5	0.946640	0.786760	0.046140	1.000000	0
O17	4	0.882980	0.762630	0.160170	1.000000	0
Fe7	5	0.894490	0.756320	0.158180	1.000000	0
O10	4	0.932500	0.817500	0.035170	0.500000	0
Fe3	5	0.941370	0.808630	0.039480	0.500000	0
H90	2	0.972980	0.825000	0.170140	1.000000	0
H91	2	0.956500	0.820730	0.169910	1.000000	0
H92	2	0.949340	0.848870	0.162230	1.000000	0
H93	2	0.965690	0.853100	0.162480	1.000000	0
H102	2	0.892020	0.836750	0.152870	1.000000	0
H103	2	0.881000	0.847770	0.146160	1.000000	0
C51	1	0.884310	0.865690	0.140320	0.500000	0
C52	1	0.890670	0.859330	0.145610	0.500000	0

C53	1	0.887970	0.850150	0.147720	1.000000	0
C54	1	0.894330	0.843790	0.151540	1.000000	0
C55	1	0.903600	0.846400	0.153370	0.500000	0
C56	1	0.910560	0.839440	0.156830	0.500000	0
C57	1	0.926010	0.835600	0.161100	1.000000	0
C58	1	0.929850	0.820150	0.165190	0.500000	0
C59	1	0.941540	0.831720	0.165010	1.000000	0
C60	1	0.951540	0.834430	0.165950	1.000000	0
C61	1	0.958400	0.827850	0.168380	1.000000	0
C62	1	0.967870	0.830300	0.168570	1.000000	0
C63	1	0.970700	0.839390	0.166320	1.000000	0
C64	1	0.963800	0.846020	0.164230	1.000000	0
C65	1	0.954380	0.843590	0.164070	1.000000	0
C66	1	0.980730	0.841820	0.165160	1.000000	0
N5	3	0.938860	0.822730	0.166410	1.000000	0
N6	3	0.935050	0.838110	0.162340	1.000000	0
N7	3	0.923410	0.826590	0.162500	0.500000	0
N8	3	0.919600	0.841970	0.158270	1.000000	0
O3	4	0.887290	0.873670	0.137970	1.000000	0
O2	4	0.987040	0.836330	0.168230	1.000000	0
O5aa	4	0.982560	0.849280	0.160820	1.000000	0
O8	4	0.875000	0.875000	0.096340	0.250000	0
H104	2	0.951370	0.777380	0.072730	1.000000	0
H105	2	0.954160	0.774590	0.089200	1.000000	0
H106	2	0.987020	0.810080	0.156220	1.000000	0
H107	2	0.978890	0.797710	0.147970	1.000000	0
H108	2	0.991980	0.806230	0.122290	1.000000	0
H109	2	1.000130	0.818530	0.130530	1.000000	0
C73	1	0.998080	0.823690	0.148700	1.000000	0
C74	1	0.994190	0.815350	0.144050	1.000000	0
C75	1	0.988290	0.809210	0.148850	1.000000	0
C76	1	0.983620	0.802120	0.144100	1.000000	0
C78	1	0.990920	0.806910	0.129720	1.000000	0
C79	1	0.995630	0.813950	0.134460	1.000000	0
C80	1	0.984690	0.801050	0.134430	1.000000	0
C81	1	0.978900	0.794280	0.129220	1.000000	0
C82	1	0.967550	0.782450	0.129180	0.500000	0
C83	1	0.972780	0.788830	0.114990	1.000000	0
C84	1	0.966080	0.783920	0.100810	0.500000	0
C85	1	0.964960	0.785040	0.090580	0.500000	0
C86	1	0.958310	0.779790	0.085680	1.000000	0
C87	1	0.956720	0.781390	0.076210	1.000000	0
C88	1	0.961740	0.788260	0.071390	0.500000	0
C89	1	0.959400	0.790600	0.061590	0.500000	0

N1	3	0.972170	0.789390	0.105580	1.000000	0
N2	3	0.978580	0.794550	0.119750	1.000000	0
N3	3	0.973350	0.788200	0.133880	1.000000	0
N4	3	0.967220	0.782780	0.119710	0.500000	0
O3aa	4	0.952900	0.786170	0.057640	1.000000	0
O19	4	0.996050	0.825260	0.157120	1.000000	0
O18	4	1.003040	0.829150	0.143920	1.000000	0
H110	2	0.920240	0.796070	0.001780	1.000000	0
H111	2	0.926760	0.801690	0.016280	1.000000	0
H112	2	0.935590	0.773800	0.023190	1.000000	0
H113	2	0.929060	0.768140	0.008620	1.000000	0
H118	2	0.912420	0.805040	-0.033780	1.000000	0
H119	2	0.912710	0.816990	-0.045730	1.000000	0
C5aa	1	0.924200	0.781660	0.003990	1.000000	0
C8	1	0.913800	0.814550	-0.064550	0.500000	0
C9	1	0.912870	0.807310	-0.057310	0.500000	0
C10	1	0.912560	0.809770	-0.047860	1.000000	0
C11	1	0.912370	0.802910	-0.041000	1.000000	0
C12	1	0.912480	0.793450	-0.043450	0.500000	0
C13	1	0.913590	0.786220	-0.036220	0.500000	0
C14	1	0.916790	0.782240	-0.020650	1.000000	0
C15	1	0.920740	0.766790	-0.016790	0.500000	0
C16	1	0.921320	0.778520	-0.005360	1.000000	0
C17	1	0.923480	0.791100	0.006420	1.000000	0
C18	1	0.928410	0.775460	0.010270	1.000000	0
C19	1	0.932170	0.778700	0.018610	1.000000	0
C20	1	0.927250	0.794340	0.014740	1.000000	0
C21	1	0.931810	0.788210	0.020830	1.000000	0
C22	1	0.936410	0.791820	0.029280	1.000000	0
N16	3	0.918470	0.784860	-0.011780	1.000000	0
N17	3	0.922430	0.769470	-0.007920	1.000000	0
N18	3	0.917970	0.773200	-0.023200	0.500000	0
N19	3	0.914640	0.788710	-0.027150	1.000000	0
O23	4	0.914240	0.822970	-0.062010	1.000000	0
O11	4	0.936720	0.800530	0.030420	1.000000	0
O12	4	0.939940	0.786080	0.034940	1.000000	0
H116	2	0.981840	0.789400	0.029410	1.000000	0
H117	2	0.990130	0.781110	0.017180	1.000000	0
C23	1	0.964850	0.785150	0.036980	0.500000	0
C24	1	0.970520	0.779480	0.030410	0.500000	0
C25	1	0.979000	0.782890	0.026910	1.000000	0
C26	1	0.983750	0.778140	0.019850	1.000000	0
C27	1	0.980160	0.769840	0.016090	0.500000	0
C28	1	0.984780	0.765220	0.008040	0.500000	0

C29	1	0.984930	0.753450	-0.003450	0.500000	0
N20	3	0.981000	0.757430	0.004170	1.000000	0
N21	3	0.992710	0.757290	-0.007290	0.166667	0
O16	4	0.956960	0.782060	0.039490	1.000000	0
O13	4	0.950410	0.799590	0.043920	0.500000	0
O20	4	0.906120	0.750000	0.156120	0.500000	0
O1	4	0.924970	0.750000	0.174970	0.500000	0
Fe6	5	0.915580	0.750000	0.165580	0.500000	0
O25	4	0.910050	0.839950	-0.057110	0.500000	0
O24	4	0.921000	0.829000	-0.079000	0.166667	0
Fe1	5	0.915470	0.834530	-0.067940	0.500000	0
H114	2	0.888050	0.841670	-0.070400	1.000000	0
H115	2	0.872040	0.845470	-0.074210	1.000000	0
C1	1	0.849540	0.849540	-0.087940	0.500000	0
C2	1	0.861510	0.838360	-0.088360	0.500000	0
C3	1	0.870770	0.835210	-0.085210	0.500000	0
C4	1	0.875410	0.839910	-0.078000	1.000000	0
C5	1	0.884610	0.837740	-0.075840	1.000000	0
C6	1	0.889380	0.830820	-0.080820	0.500000	0
C7	1	0.899330	0.829020	-0.079020	0.500000	0
N22	3	0.857480	0.845940	-0.084380	1.000000	0
N23	3	0.845590	0.845590	-0.095590	0.166667	0
O26	4	0.903400	0.833810	-0.072850	1.000000	0
